# Radon signature of CO_2_ flux constrains the depth of degassing: Furnas volcano (Azores, Portugal) versus Syabru-Bensi (Nepal Himalayas)

**DOI:** 10.1038/s41598-022-14653-5

**Published:** 2022-06-27

**Authors:** Frédéric Girault, Fátima Viveiros, Catarina Silva, Sandeep Thapa, Joana E. Pacheco, Lok Bijaya Adhikari, Mukunda Bhattarai, Bharat Prasad Koirala, Pierre Agrinier, Christian France-Lanord, Vittorio Zanon, Jean Vandemeulebrouck, Svetlana Byrdina, Frédéric Perrier

**Affiliations:** 1 Institut de Physique du Globe de Paris, CNRS, Université Paris Cité, 75005 Paris, France; 2 Instituto de Investigação em Vulcanologia e Avaliação de Riscos (IVAR), Universidade dos Açores, Ponta Delgada, Portugal; 3 Faculdade de Ciências e Tecnologia, Universidade dos Açores, Ponta Delgada, Portugal; 4 Centro de Informação e Vigilância Sismovulcânica dos Açores, Ponta Delgada, Portugal; 5 Department of Mines and Geology, National Seismological Centre, Lainchaur, Kathmandu, Nepal; 6 Centre de Recherches Pétrographiques et Géochimiques, Université de Lorraine-CNRS, 54500 Vandoeuvre-lès-Nancy, France; 7 CNRS, IRD, IFSTTAR, ISTerre, Université Savoie Mont Blanc, 73000 Chambéry, France

**Keywords:** Geochemistry, Volcanology, Natural hazards, Solid Earth sciences

## Abstract

Substantial terrestrial gas emissions, such as carbon dioxide (CO_2_), are associated with active volcanoes and hydrothermal systems. However, while fundamental for the prediction of future activity, it remains difficult so far to determine the depth of the gas sources. Here we show how the combined measurement of CO_2_ and radon-222 fluxes at the surface constrains the depth of degassing at two hydrothermal systems in geodynamically active contexts: Furnas Lake Fumarolic Field (FLFF, Azores, Portugal) with mantellic and volcano-magmatic CO_2_, and Syabru-Bensi Hydrothermal System (SBHS, Central Nepal) with metamorphic CO_2_. At both sites, radon fluxes reach exceptionally high values (> 10 Bq m^−2^ s^−1^) systematically associated with large CO_2_ fluxes (> 10 kg m^−2^ day^−1^). The significant radon‒CO_2_ fluxes correlation is well reproduced by an advective–diffusive model of radon transport, constrained by a thorough characterisation of radon sources. Estimates of degassing depth, 2580 ± 180 m at FLFF and 380 ± 20 m at SBHS, are compatible with known structures of both systems. Our approach demonstrates that radon‒CO_2_ coupling is a powerful tool to ascertain gas sources and monitor active sites. The exceptionally high radon discharge from FLFF during quiescence (≈ 9 GBq day^−1^) suggests significant radon output from volcanoes worldwide, potentially affecting atmosphere ionisation and climate.

## Introduction

Since decades, the tenuous relation between Earth’s deformation, earthquakes, and carbon dioxide (CO_2_) release has fostered broad interest in the geoscience’s community^1,2^. Significant CO_2_ emissions have been commonly reported at the plate boundaries in a variety of seismotectonic regimes: extension (rifting)^3^, reverse fault^4^, strike-slip fault^5^, subduction^6^, triple junction^7^, and collision^8^. To appreciate the potential coupling between geodynamics and CO_2_, we need to better understand CO_2_ sources and transport mechanisms. Besides biological sources, the released geogenic CO_2_ generally has either a volcano-magmatic, a mantellic or a metamorphic source, or a mixing of these. The number of available data-sets on CO_2_ release has increased^9^, and it appears timely to evaluate the available results against each other. Comparing CO_2_-emitting sites in different active tectonic settings may help diagnose the involved gas transport mechanisms and constrain the depth of degassing (i.e., gas source depth). Both are delicate questions at all sites, but appear fundamental for long-term monitoring, prediction, and health risk assessment of the population. For this purpose, coupling CO_2_ measurement with that of a trace gas, such as helium, mercury or radon, may reveal of utmost value.

Radon-222, a noble, alpha-emitter radioactive gas with half-life of 3.8 days, is produced in the upper crust by alpha-decay of the solid radium-226. Tagged as a tracer of fluid transport at several active faults and hydrothermal systems worldwide, it was found sensitive to deformation and earthquakes, both in the field^10,11^ and laboratory^12,13^, although many claimed precursory signals remain controversial^14^. To migrate from its source to the surface before decaying, radon needs to be transported by a carrier fluid having a sufficient velocity, such as CO_2_, at high-permeability geosystems. Indeed, the use of radon to constrain gas transport and source has been pioneered at a few CO_2_-emitting sites. For example, at Santorini volcano (Greece), carbon isotopic composition was compared with radon concentration, but without accounting for heterogeneity of the layers crossed by the fluid mixture^15^. At Mt. Etna volcano (Italy), radon transport was modelled through geological layers, but without accounting for production terms within the layers^16^. At Pantelleria Island (Italy) and Nisyros volcano (Greece), ^222^Rn/^220^Rn concentration ratios and few radon source term values in soils and rocks were used, but without radon transport modelling^17,18^. To infer constraints on gas source and transport mechanisms by coupling CO_2_ and radon measurements, a radon transport model, together with a thorough characterisation of radon sources, is necessary to interpret the data.

Here, we apply a combined approach of coupling the measurements of CO_2_ flux and radon flux from the ground surface to constrain the depth of degassing at two geodynamically active sites with different CO_2_ sources: Furnas Lake Fumarolic Field (FLFF), São Miguel Island, Azores archipelago, Portugal with mantellic and volcano-magmatic CO_2_ sources, and Syabru-Bensi Hydrothermal System (SBHS) in the Himalayas of Central Nepal with a metamorphic CO_2_ source. We show that the combination of radon flux, CO_2_ flux, radon sources, and CO_2_ sources, interpreted using a common simplified multiphase radon transport model, constrains the depth of CO_2_ degassing at both volcanic (FLFF) and active fault sites (SBHS), and allows quantifying deep-originated gas fluxes to the atmosphere.

**Figure 1:**
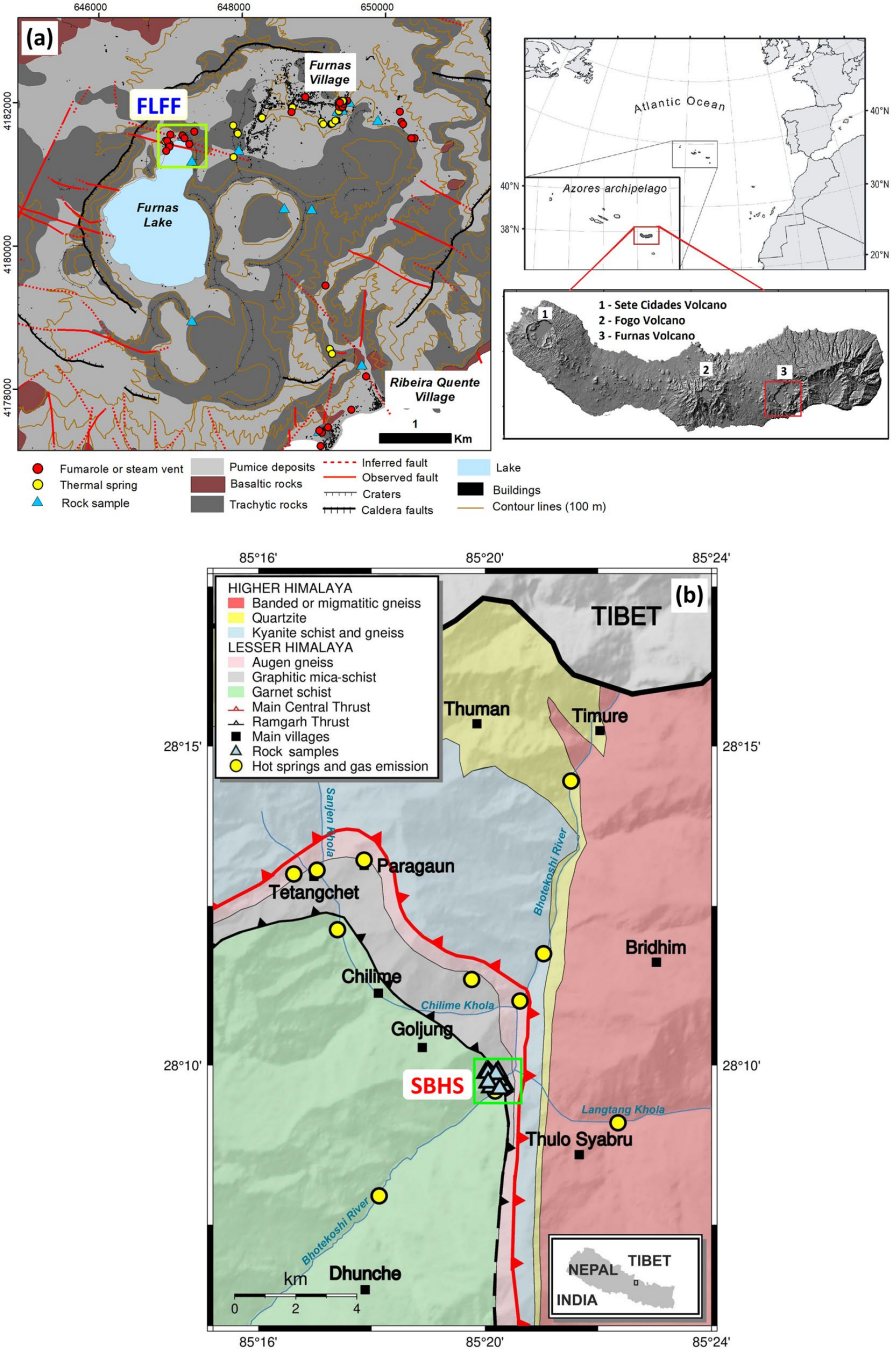
**(a)** Geological map showing the location of the Furnas Lake Fumarolic Field (FLFF) in mantellic and volcano-magmatic context (Azores, Portugal). The insets show the location of the Azores and of the Furnas Volcano on the São Miguel Island. (**b**) Geological map showing the location of the Syabru-Bensi Hydrothermal System (SBHS) in metamorphic and active tectonic context (Central Nepal). The inset shows the location in the Nepal map.

The Azores archipelago, formed by nine volcanic islands along a WNW-ESE trend, is located in the North Atlantic Ocean at the triple junction of the American, Eurasian, and Nubian tectonic plates. The largest island, São Miguel, comprises three dormant trachytic polygenetic volcanoes (from west to east: Sete Cidades, Fogo, and Furnas)^19^. Furnas volcano (Fig. 1a), with an age of approximately 100,000 years B.P., is formed by two nested calderas controlled by a NW-SE and NE-SW trending fault system^20^. The western part of the youngest caldera is filled by a shallow (≤ 15 m depth) lake. Several eruptive styles occurred at Furnas volcano, from effusive to caldera-forming explosive events, with a total of 10 intra-caldera eruptions in the last 5000 years^21^. Since the fifteenth century, two major eruptions have occurred: one event in 1439‒1443 and one deadly subplinian event in 1630. The erupted products were mainly pumices, ashes and lapilli, surges and trachytic lava domes^20^.

The current volcanic activity takes the form of secondary manifestations, such as boiling fumaroles, thermal and cold CO_2_-rich springs, and CO_2_ diffuse degassing structures (DDS). One of the main degassing areas (Fig. 1a) is Furnas Lake Fumarolic Field (FLFF) located north of Furnas Lake^22^. Gas released is dominated by water steam and CO_2_, with detectable traces of H_2_S, H_2_, N_2_, CH_4_, Ar, He, and CO^22^. According to the recently published conceptual model, the released gases come from the final cooling of a trachytic reservoir located around 3‒4 km depth^23^, percolate through the fracture network, dissolve in shallow meteoric aquifers with vapour/liquid equilibrium temperature around 270 °C in Furnas Lake area, degas and then percolate to the surface^22,24^. This is consistent with various geophysical soundings performed at FLFF, which showed P-wave low velocity zone at 6 km depth^25^, low density magma bodies at 4‒5 km depth^26^, and conductive zones at 500 m and 100 m depth^27^ dipping below the lake^28^. Chemistry and isotopic composition of thermal waters and fumaroles at FLFF^7,22,29,30^ suggest mantellic and volcano-magmatic CO_2_ (δ^13^C = − 4.5 ± 0.2 ‰; *n* = 3) and a mixing of mantellic and crustal components for helium (*R*/*R*_a_ = 5.25 ± 0.02; *n* = 3).

The Azores climate is oceanic temperate, with a mean annual temperature of 17 °C (minimum of 14 °C in January and maximum of 25 °C in August). The mean annual precipitation of 1930 mm year^−1^ is marked by a strong seasonality between a rainy season from October to March (75% of annual precipitation) and a dry season in summer^31^. At FLFF, CO_2_ is released by fumaroles, DDS, and thermal springs. Several CO_2_ flux studies^7,32^ have been carried out at the four main CO_2_ emitting areas of Furnas volcano (FLFF, Furnas Village, Ribeira dos Tambores, and Ribeira Quente Village). Recent total estimated gaseous CO_2_ discharge from FLFF^33^ amounts to 35 t day^−1^, among which DDS represent^33,34^ 6.0 ± 0.2 t day^−1^ and 19.8 t day^−1^, respectively over 4000 m^2^ and 20,000 m^2^ surface area. In the FLFF surroundings, a permanent CO_2_ flux station is operating since 2004^32,35^. Potential radon sources may be associated with ^226^Ra excess in basaltic lavas^36^, ^226^Ra secondary mineralisation within ashes, pumices, lapilli, and altered soil surface, and ^226^Ra dissolved in thermal waters. Large radon concentrations are reported in the ground (max: 390,000 Bq m^−3^) and in habitations (max: 13,300 Bq m^−3^) at Furnas volcano^37,38^, which, together with CO_2_, pose substantial health hazard to the population^7,39^.

The second site considered in this paper is located in the Nepal Himalayas. The Himalayan orogen results from the India–Eurasia collision which started 55 Ma ago. Half of the continent–continent shortening, 2 cm year^−1^, is accommodated by the large décollement called the Main Himalayan Thrust (MHT)^40^. In Nepal, the last large earthquake, the 2015 *M*_w_7.9 Gorkha earthquake, claiming > 9000 lives, ruptured the MHT over 150 km^41^. About 4–5 events of local magnitude *M*_L_ > 5 occur per year, concentrated at 10–25 km depth, at the foot of the Himalayan topographic rise^42^. This area, the Main Central Thrust (MCT) zone^43^, is a 2- to-10-km-width shear zone that separates high-grade meta-crystalline rocks from the Greater Himalaya to the north from low-grade meta-sedimentary rocks from the Lesser Himalaya to the south.

Along the whole Himalayas, the MCT zone comprises numerous hydrothermal systems characterised by thermal and cold CO_2_-rich springs^44,45^, ‘tectonic’ fumaroles, and CO_2_ DDS^46^, with significant CO_2_ emissions concentrated along a 110-km-long segment in Central Nepal^47^. The upper Trisuli valley (Fig. 1b), located 60 km north to Kathmandu, encompasses several CO_2_-emitting hydrothermal sites in the vicinity of the MCT zone and shows the largest Himalayan CO_2_ release reported so far (> 15 ± 3 t day^−1^)^47,48^. This valley also reported significant post-seismic hydrothermal changes following the Gorkha earthquake^8^. One of these sites, Syabru-Bensi Hydrothermal System (SBHS) (Fig. 1b), is located 3.5 km south to MCT within the Lesser Himalayan rocks comprising mainly mica-schist, quartzite, marble, graphitic schist, and augen gneiss^49–51^. Gas released is dominated by CO_2_, with water steam and large H_2_S content (340 ppm). According to the most recent conceptual model^8^, CO_2_-rich gas comes from a deep source at > 5 km depth, percolates through fracture networks in the MCT zone, forms gas reservoirs and mixes with meteoric water, degasses at a depth of 100 m or more at 70 °C ≤ *T* ≤ 120 °C (vapour/liquid equilibrium temperature), and then crosses near-surface aquifers before reaching the surface. This appears consistent with geophysical soundings carried out at SBHS and in the valley which showed a highly conductive and low P-wave velocity zone at ≈ 10 km depth^52^, conductive zones at 10‒30 m depth and shallow altered fractured conduits for gas below the SBHS^53^, and self-potential anomalies at the surface^53,54^. Chemistry and isotopic composition of thermal waters and DDS at SBHS^8,44,45,54^ suggest CO_2_ production by metamorphic decarbonation at > 5 km depth, a dominating crustal carbon component of the gaseous CO_2_ (δ^13^C = − 0.75 ± 0.01‰; *n* = 27), and radiogenic helium (*R*/*R*_a_ ≤ 0.05; *n* = 2).

Circumvented respectively to the west, north, and east by Ganesh, Tibet, and Langtang ranges, SBHS benefits from a rain shadow effect of Gosainkunda range to the southeast. The mean annual precipitation varies from 1100 to 1800 mm year^−1^. Monsoon occurs from June to September (80% of annual precipitation) and dry season from December to February^55^. The mean annual air temperature is 19 °C, with minimum of 0 °C in January and maximum of 28 °C in June. At SBHS, CO_2_ is released by fumaroles, DDS, and thermal springs. Several CO_2_ flux studies^8,46,47,54,56^ have been carried out at the five main CO_2_-emitting areas of SBHS. Total estimated gaseous CO_2_ discharge from SBHS^8,47,54^ amounts to 8 t day^−1^, among which 3.8 ± 0.4 t day^−1^ corresponds to DDS of gas zones 1 and 2 over a surface area of 11,000 m^2^. At this site, several radon measurement campaigns reported large radon fluxes at the surface (max: 38,500 × 10^−3^ Bq m^−2^ s^−1^) and radon concentrations in the ground (max: 57,700 Bq m^−3^)^8,46,54,57^. Radon sources have also been investigated in the thermal and cold waters, the soil, and the surrounding rock layers^50,54,58^. These high CO_2_ and radon releases also pose a health hazard to the local population and animals^54,57^.

## Results

### Radon and CO_2_ fluxes at Furnas Fumarolic Field

Radon and CO_2_ fluxes were measured in June 2016 at the surface using the accumulation chamber method (see “Methods” section; Supp. Fig. S1) around the main boiling fumaroles and near the lakeshore. A total of 169 radon and 371 CO_2_ flux measurements were performed at 136 and 335 locations (Table 1), respectively, over a surface area of 48,000 m^2^. Fluxes were measured along several profiles every 2 to 5 m (Supp. Fig. S2).

**Table 1:** Overview of radon and CO_2_ flux data-set separately at Furnas Lake Fumarolic Field (FLFF) and Syabru-Bensi Hydrothermal System (SBHS).

Site	FLFF	SBHS
**Radon-222 flux (10**^**−3**^** Bq m**^**−2**^** s**^**−1**^**)**		
Investigated surface area (m^2^)	44,350	948
Number of measurements	169	418
Number of measured points	136	250
Min–max range	1.34–39,558	1.01–20,476
Arithmetic mean	4302 ± 636	1142 ± 139
Geometric mean	562 ± 13	233.6 ± 3.4
Median (at 90% CL)	722 ± 18	225.8 ± 9.9
Min–max range (at 90% CL)	6.6–22,780	12.1–4999
Normal probability partitioning		
Population A: min–max (fraction)	1.3–870 (54.7%)	1.0–130 (43.0%)
Population B: min–max (fraction)	870–40,000 (45.3%)	130–20,000 (57.0%)
Estimated total discharge (MBq d^−1^)	9300 ± 1600	71 ± 11
Estimated total discharge (Bq s^−1^)	108,000 ± 19,000	820 ± 130
Estimated surface of flux > 1000 × 10^−3^ Bq m^−2^ s^−1^ (m^2^)	23,864	172
Estimated surface of flux > 500 × 10^−3^ Bq m^−2^ s^−1^ (m^2^)	32,532	411
Estimated surface of flux > 300 × 10^−3^ Bq m^−2^ s^−1^ (m^2^)	36,405	589
Estimated surface of flux > 100 × 10^−3^ Bq m^−2^ s^−1^ (m^2^)	41,118	856
Estimated surface of flux > 22 × 10^−3^ Bq m^−2^ s^−1^ (m^2^)	43,654	937
**CO**_**2**_ **flux (g m**^**−2**^ **day**^**−1**^**)**		
Investigated surface area (m^2^)	47,745	3078
Number of measurements	371	777
Number of measured points	335	399
Min–max range	8.1–26,698	2.76–155,178
Arithmetic mean	1227 ± 184	6463 ± 1007
Geometric mean	139.4 ± 0.9	284.0 ± 2.7
Median (at 90% CL)	83.3 ± 0.2	173.6 ± 1.4
Min–max range (at 90% CL)	15.8–7126	10.1–46,773
Normal probability partitioning		
Population A: min–max (fraction)	8.1–720 (80.8%)	2.8–260 (54.8%)
Population B: min–max (fraction)	720–27,000 (19.2%)	260–7800 (33.9%)
Population C: min–max (fraction)		7800–160,000 (11.3%)
Estimated total discharge (t day^−1^)	19.1 ± 5.3	6.3 ± 1.6
Estimated total discharge (mol s^−1^)	5.0 ± 1.4	1.65 ± 0.41
Estimated surface of flux > 1000 g m^−2^ day^−1^ (m^2^)	3638	890
Estimated surface of flux > 500 g m^−2^ day^−1^ (m^2^)	4070	1239
Estimated surface of flux > 300 g m^−2^ day^−1^ (m^2^)	4164	1536
Estimated surface of flux > 100 g m^−2^ day^−1^ (m^2^)	24,428	2178
Estimated surface of flux > 25 g m^−2^ day^−1^ (m^2^)	45,192	2810
Estimated surface of flux > 10 g m^−2^ day^−1^ (m^2^)	47,743	3045
**Radon–CO**_**2**_ **fluxes correlation**		
Number of averaged values	136	157
Correlation coefficient	0.83 ± 0.02	0.80 ± 0.03

Estimated surface discharge of radon and CO_2_ are also given for both sites. All experimental uncertainties are given at one standard deviation (1-σ, 68% confidence level).

**Figure 2:**
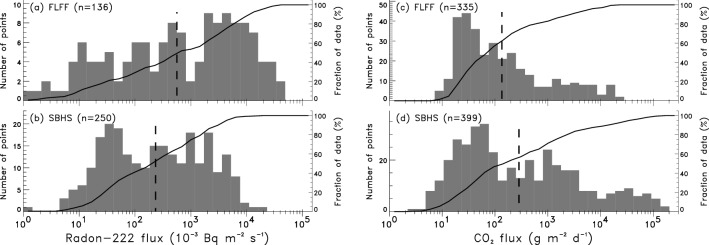
Distributions of radon and CO_2_ fluxes measured at both sites (in logarithmic scale): on the left, radon flux at **(a)** FLFF and **(b)** SBHS; on the right, CO_2_ flux at **(c)** FLFF and **(d)** SBHS. Geometric mean of each distribution is represented as a vertical dashed black line and the cumulated distribution as a solid black curve (scale on the right-hand side).

Mean radon flux values range over five orders of magnitude from 1.3 to 40,000 × 10^−3^ Bq m^−2^ s^−1^ (Fig. 2a). The largest radon fluxes (> 10,000 × 10^−3^ Bq m^−2^ s^−1^) are found in the fumarolic area. Such huge values are commonly reported on uranium mill tailings (see review^54^). The overall arithmetic (geometric) mean for radon fluxes amounts to 4300 ± 600 (560 ± 10) × 10^−3^ Bq m^−2^ s^−1^, which is more than three times larger than the few reported mean radon flux values obtained at volcanic sites worldwide (see review^54^). The distribution of radon flux values is bimodal (Fig. 2a), with populations A (55%) and B (45%) separated by a threshold value of 870 × 10^−3^ Bq m^−2^ s^−1^ using normal probability partitioning^59^ (Table 1); the median (720 ± 20 × 10^−3^ Bq m^−2^ s^−1^) is larger than the mean. Mean CO_2_ flux values range over five orders of magnitude from 8.1 to 27,000 g m^−2^ day^−1^ (Fig. 2c), with the largest values (> 1000 g m^−2^ day^−1^) also found in the fumarolic area. The overall arithmetic (geometric) mean for CO_2_ fluxes amounts to 1200 ± 200 (139 ± 1) g m^−2^ day^−1^, compatible with past measurement campaigns^7,33,34^. The distribution of CO_2_ flux values is bimodal, with populations A (81%) and B (19%) separated by a threshold value of 720 g m^−2^ day^−1^ (Table 1); the median (83.3 ± 0.2 g m^−2^ day^−1^) is smaller than the mean.

**Figure 3:**
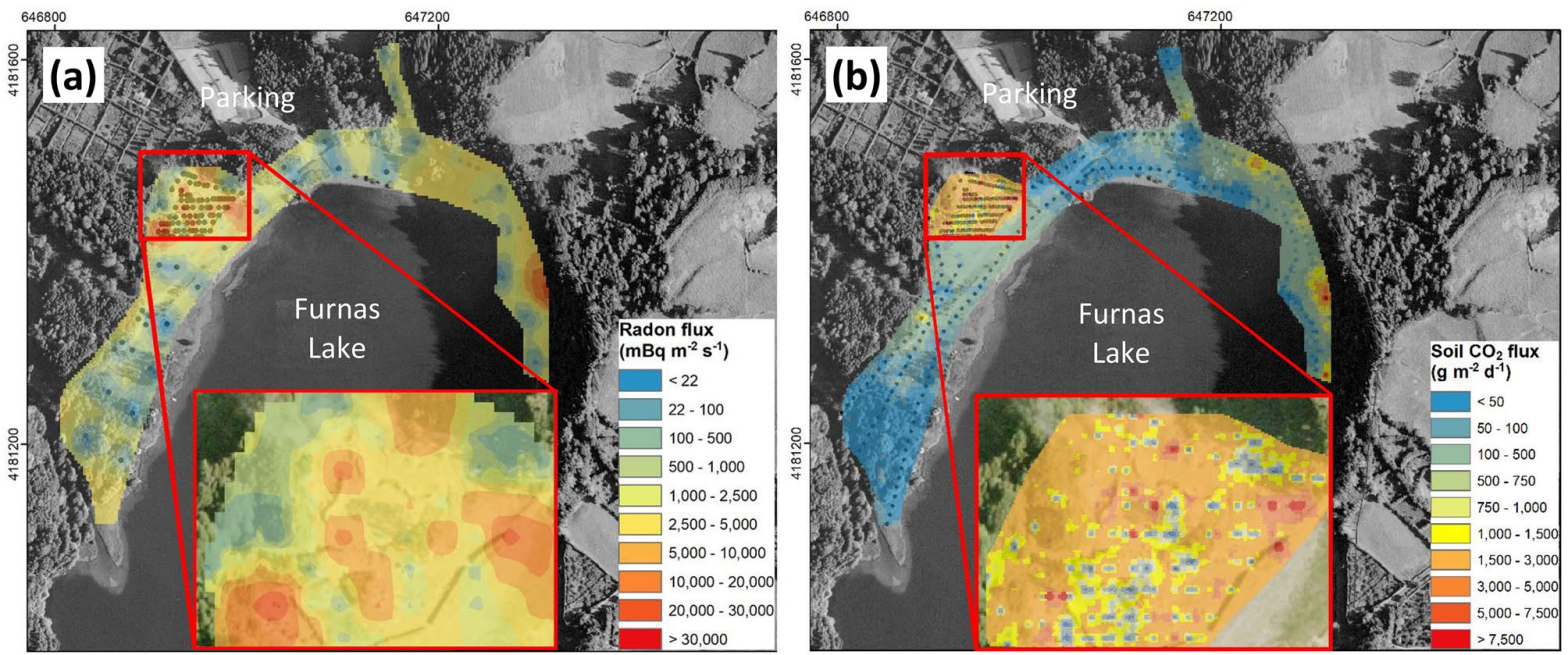
Interpolated **(a)** radon and **(b)** CO_2_ flux maps of FLFF. For each flux map, the colour scale is shown on the bottom right. In **(a,b)**, the inset is an enlargement of the fumarolic area. The map is projected following the UTM coordinate system. The map was built using sGs method with a cell size of 3 m^2^ (see “Methods” section).

Based on the whole flux data-set and using sequential Gaussian simulations (see “Methods” section), maps of radon and CO_2_ fluxes are obtained at FLFF (Fig. 3). The largest radon fluxes are concentrated in the fumarolic area, near the parking, and on the eastern part, while the lowest are found in the western lakeshore and between the fumarolic area and the parking (Fig. 3a). Significant spatial variations of radon flux are found within the fumarolic area over an area of 80 × 100 m^2^ (inset of Fig. 3a), with difference of several orders of magnitude within few metres only. On the eastern part, large radon fluxes appear isolated. The largest CO_2_ fluxes are also concentrated in the fumarolic area, near the parking and on the eastern part, while the lowest are found in the western lakeshore and between the fumarolic area and the parking (Fig. 3b). Significant spatial variations of CO_2_ flux, larger than for radon fluxes, are found within the fumarolic area (inset of Fig. 3b), with difference of several orders of magnitude within few metres only. On the eastern and western parts and near the parking, large CO_2_ fluxes reflect areas with a higher mean CO_2_ flux. The surface area of large CO_2_ flux is larger than that of large radon flux.

Based on our data-set, past CO_2_ flux campaigns^7^, and the literature, mean background fluxes are estimated as 22 × 10^−3^ Bq m^−2^ s^−1^ for radon and 25 g m^−2^ day^−1^ for CO_2_. The surface areas of radon and CO_2_ fluxes above background yield 43,700 and 45,200 m^2^, respectively (about 97% of the investigated surface area). Exceptionally large radon (> 1000 × 10^−3^ Bq m^−2^ s^−1^) and CO_2_ fluxes (> 1000 g m^−2^ day^−1^) occupy a surface area of 23,900 m^2^ (54%) and 3600 m^2^ (8%), respectively. The total estimated radon discharge amounts to 9300 ± 1600 MBq day^−1^ (108,000 ± 19,000 Bq s^−1^). To date, this huge estimate is the first obtained at a volcanic hydrothermal system. The total estimated CO_2_ discharge amounts to 19.1 ± 5.3 t day^−1^ (5.0 ± 1.4 mol s^−1^). This value, consistent with reported CO_2_ discharges obtained during past campaigns^7,34^, is similar to other volcanic sites worldwide^47^.

### Radon and CO_2_ fluxes at Syabru–Bensi hydrothermal system

Radon and CO_2_ fluxes were regularly measured from 2009 to 2020 at the surface using the accumulation chamber method (see “Methods” section; Supp. Fig. S1) around the tectonic fumaroles and non-vegetated areas, 20 m above the main hot springs (gas zones 1‒2). A total of 418 radon and 777 CO_2_ flux measurements were performed at 250 and 399 locations (Table 1), respectively, over a surface area of 3000 m^2^. Fluxes were measured along several profiles every 1 to 2 m (Supp. Fig. S3).

Mean radon flux values range over five orders of magnitude from 1.0 to 20,000 × 10^−3^ Bq m^−2^ s^−1^ (Fig. 2b). The largest radon fluxes (> 10,000 × 10^−3^ Bq m^−2^ s^−1^) are concentrated near the tectonic fumaroles. The overall arithmetic (geometric) mean for radon fluxes amounts to 1100 ± 100 (234 ± 3) × 10^−3^ Bq m^−2^ s^−1^. The distribution of radon flux values is bimodal (Fig. 2b), with populations A (43%) and B (57%) separated by a threshold value of 130 × 10^−3^ Bq m^−2^ s^−1^ (Table 1); the median (230 ± 10 × 10^−3^ Bq m^−2^ s^−1^) is similar to the mean. Mean CO_2_ flux values range over six orders of magnitude from 2.8 to 160,000 g m^−2^ day^−1^ (Fig. 2d). The largest CO_2_ fluxes (> 1000 g m^−2^ day^−1^) are also measured in the vicinity of the fumaroles. The overall arithmetic (geometric) mean for CO_2_ fluxes amounts to 6500 ± 1000 (284 ± 3) g m^−2^ day^−1^. The distribution of CO_2_ flux values bears three modes (Fig. 2d), with populations A (55%), B (34%) and C (11%) separated by threshold values of 260 and 7800 g m^−2^ day^−1^, respectively (Table 1); the median (174 ± 1 g m^−2^ day^−1^) is smaller than the mean.

**Figure 4:**
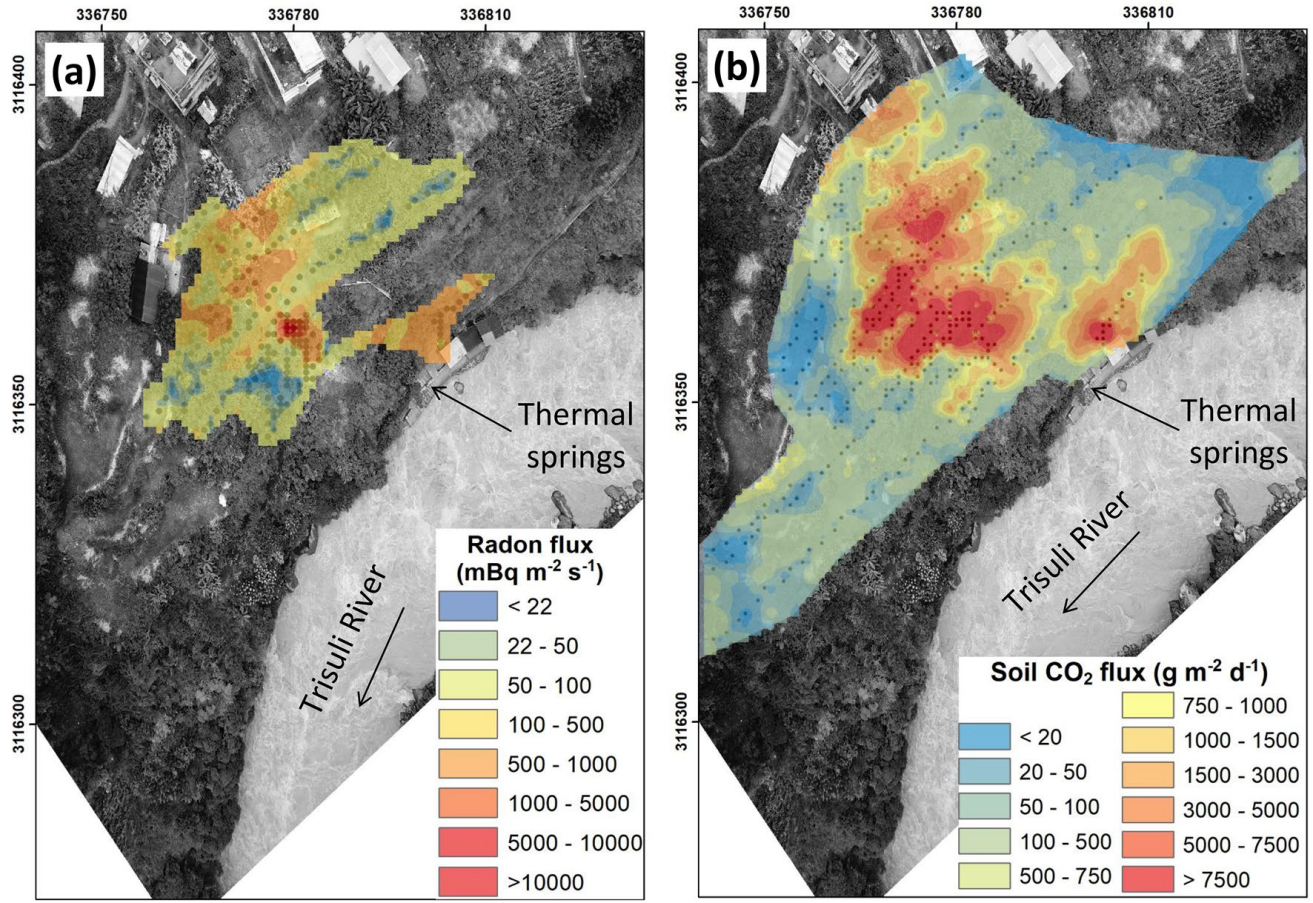
Interpolated **(a)** radon and **(b)** CO_2_ flux maps of SBHS. For each flux map, the colour scale is shown on the bottom right. The map is projected following the UTM coordinate system. The map was built using sGs method with a cell size of 1 m^2^ (see “Methods” section).

Similarly (see “Methods” section), maps of radon and CO_2_ fluxes are obtained at SBHS (Fig. 4). The largest radon fluxes are concentrated in the vicinity of small recesses at the base of terrace scarps, in non-vegetated areas, and at the foot of the terraces arranged for crops, while the lowest are found in the southern and northern parts of the alluvial and debris fall terrace (Fig. 4a). Here also, metre-scaled variations of radon flux of several orders of magnitude are noticed around recesses and non-vegetated areas over a surface area of 30 × 45 m^2^ (Fig. 4a). In the western area, large radon fluxes are found in the vicinity of inhabited housings. The largest CO_2_ fluxes are also concentrated inside and near the recesses, in non-vegetated areas, and at the foot of terraces, while the lowest are found in the southern and northern parts (Fig. 4b). Similarly, significant spatial variations of CO_2_ flux are found near the recesses and non-vegetated areas (Fig. 4b), with difference of several orders of magnitudes within few metres only. Here also, the surface area of large CO_2_ flux is larger than that of large radon flux.

Based on the overall data-set, mean background fluxes are estimated as 22 × 10^−3^ Bq m^−2^ s^−1^ for radon and 10 g m^−2^ day^−1^ for CO_2_. The surface areas of radon and CO_2_ fluxes above background yield 940 m^2^ and 3000 m^2^, respectively (about 99% of the investigated surface area). Exceptionally large radon (> 1000 × 10^−3^ Bq m^−2^ s^−1^) and CO_2_ fluxes (> 1000 g m^−2^ day^−1^) occupy a surface area of 170 m^2^ (18%) and 890 m^2^ (29%), respectively. The total estimated radon discharge amounts to 71 ± 11 MBq day^−1^ (820 ± 130 Bq s^−1^). The total estimated CO_2_ discharge amounts to 6.3 ± 1.6 t day^−1^ (1.65 ± 0.41 mol s^−1^), similar to previously published values at this site^8,54^ and to other mofette sites worldwide^47^.

### Radon sources

At FLFF and SBHS, numerous potential sources of radon were investigated (Table 2). At FLFF, 45 soil and 18 rock samples were analysed for their effective radium-226 concentration (*EC*_Ra_, or radon source term; see “Methods” section). Soil samples consist mostly of altered surface rich in kaolinite and marcasite^29^ in the fumarolic area (*n* = 30), and of pumice volcanic material of the latest explosive eruption^35^ outside (*n* = 15). Soil *EC*_Ra_ values range from 2.3 to 21 Bq kg^−1^, with an arithmetic (geometric) mean of 8.6 ± 0.7 (7.4 ± 0.1) Bq kg^−1^ (Supp. Fig. S4). This value is consistent with the mean *EC*_Ra_ value for soils^60,61^ (≈ 7 Bq kg^−1^; *n* = 2070). *EC*_Ra_ values for volcanic rocks from samples collected inside and around the Furnas caldera, i.e. altered rock and pumice (lapilli and ash), range from 0.3 to 34 Bq kg^−1^, with arithmetic (geometric) mean of 6.2 ± 2.0 (3.2 ± 0.1) Bq kg^−1^ (Supp. Fig. S4), significantly larger than the mean *EC*_Ra_ value for volcanic rocks^60^ (1.7 ± 0.2 Bq kg^−1^; *n* = 349; Supp. Fig. S5a), suggesting a crustal signature.

**Table 2:** Overview of radon and CO_2_ sources data-set separately at FFLF and SBHS.

Site		FLFF	SBHS
**Radon-222 sources**			
Soil *EC*_Ra_ (Bq kg^−1^)	Number of soil samples	45	68
	Min–max range	2.30–20.9	1.03–42.9
	Min–max range (at 90% CL)	2.80–16.7	2.02–15.8
	Arithmetic mean	8.56 ± 0.69	9.15 ± 0.72
	Geometric mean	7.44 ± 0.07	7.68 ± 0.06
Rock *EC*_Ra_ (Bq kg^−1^)	Number of rock samples	18	19
	Min–max range	0.27–33.6	0.13–7.46
	Min–max range (at 90% CL)	0.27–33.6	0.13–7.46
	Arithmetic mean	6.2 ± 2.0	2.47 ± 0.49
	Geometric mean	3.24 ± 0.05	1.55 ± 0.08
Ground gas radon-222 concentration (Bq m^−3^)	Number of sample points	86	6
	Min–max range	179–376,500	39,000–48,700
	Min–max range (at 90% CL)	1530–152,000	39,000–48,700
	Mean ± 1σ	33,106 ± 7246	43,017 ± 1795
Water bubbling radon-222 concentration (Bq m^−3^)	Number of water bubbling samples	3	2 (other springs)
	Range min–max	43,150–105,361	9137–13,559
	Mean ± 1σ	74,300 ± 18,000	11,348 ± 1563
Water radium-226 concentration (10^−3^ Bq L^−1^)	Number of springs	2	2
	Range min–max	19–107	32–218
	Mean ± 1σ	63 ± 32	125 ± 66
Water radon-222 concentration (Bq L^−1^)	Number of springs	2	2
	Range min–max	3.9–4.3	2.6–27.3
	Mean ± 1σ	4.10 ± 0.18	15.1 ± 8.8
**CO**_**2**_ **sources**			
Ground gas CO_2_ concentration (%)	Number of sample points	86	4
	Min–max range	0.3–99.9	90.0–98.1
	Min–max range (at 90% CL)	0.7–99.9	90.0–98.1
	Mean ± 1σ	56.8 ± 4.3	95.4 ± 1.9
δ^13^C of gaseous CO_2_ (‰)	Number of sample points	6^a^	4
	Range min/max	− 4.80/3.32	− 0.768/–0.713
	Mean ± 1σ	− 4.35 ± 0.22	− 0.746 ± 0.011
Dissolved inorganic carbon content (10^−3^ mol L^−1^)	Number of water springs	1^b^	2
	Range min–max		29.58–32.26
	Mean ± 1σ	9.0 ± 0.9	30.92 ± 0.95
δ^13^C of dissolved CO_2_ (‰)	Number of water springs	1^b^	2
	Range min/max		− 0.063/0.968
	Mean ± 1σ	*ca.* − 4	0.45 ± 0.36
**Gas temperature**			
Surface temperature (°C)	Number of sample points	335	31^c^
	Min–max range	17.3–98.3	8.2–24.7
	Min–max range (at 90% CL)	18.2–52.5	8.2–24.7
	Mean ± 1σ	29.55 ± 0.75	16.31 ± 0.77
Ground temperature (°C)	Number of sample points	85	8
	Min–max range	17.1–99.4	21.6–28.8
	Min–max range (at 90% CL)	18.1–97.9	21.6–28.8
	Mean ± 1σ	45.8 ± 3.0	25.78 ± 0.87

Surface and ground gas temperature is also given for both sites.

^a^Include four original data, one value from Ref.^7 ^and one value from Ref.^22^.

^b^Data from Ref.^24^.

^c^Inferred from surface temperature data used to estimate surface heat fluxes from Ref.^54^.

Radon concentration in ground gas, measured at 86 locations (see “Methods” section), ranges from 179 to 377,000 Bq m^−3^, with a mean of 33,100 ± 7200 Bq m^−3^, consistent with reported values^37,38^ and mean radon concentration in water bubbling (74,000 ± 18,000 Bq m^−3^). By contrast, radon concentration in the boiling fumarole is low (810 ± 31 Bq m^−3^). Mean radium-226 and radon concentrations in thermal water (see “Methods” section) are low (means of 63 ± 32 × 10^−3^ Bq L^−1^ and 4.1 ± 0.2 Bq L^−1^, respectively), in the lower range of values for hydrothermal waters^62^.

At SBHS, 68 soil and 19 rock samples give arithmetic mean (min‒max) *EC*_Ra_ values of 9.2 ± 0.7 (1.0‒43) and 2.5 ± 0.5 (0.13‒7.5) Bq kg^−1^, respectively (Supp. Fig. S4). Soil consists of debris fall deposit of mica-schist mixed with alluvial soil in the vegetated area, and of hydrothermal soil (“reduktosol” or “mofettic” qualification^63^) richer in organic matter, clay, and secondary iron oxides^54^ in the non-vegetated area. The metamorphic rocks are mainly garnet-rich mica-schist, marble, and quartzite. Their *EC*_Ra_ values are relatively similar to the mean *EC*_Ra_ value for metamorphic rocks (5.1 ± 0.4 Bq kg^−1^; *n* = 1256; Supp. Fig. S5b). Mean soil *EC*_Ra_ is similar to that obtained at FLFF, but mean rock *EC*_Ra_ is smaller.

Radon concentration in ground gas, measured at six locations, yields mean of 43,000 ± 1800 Bq m^−3^. Bubbling waters, only reported on the opposite river bank, give smaller radon concentration in water bubbles (mean: 11,300 ± 1600 Bq m^−3^). Similarly, mean radium-226 and radon concentrations in thermal waters are not exceptional (means of 125 ± 66 × 10^−3^ Bq L^−1^ and 15 ± 9 Bq L^−1^, respectively).

### CO_2_ sources

At FLFF, ground gas CO_2_ concentration, measured at 89 locations (see “Methods” section), ranges from 0.3 to ≈ 100 vol% (mean: 57 ± 4%), consistent with reported values^7,39^. Total dissolved inorganic carbon (DIC) concentration in thermal waters gives 9.0 ± 0.9 × 10^−3^ mol L^−1^. The carbon isotopic composition of CO_2_, with mean δ^13^C of − 4.2 ± 0.3‰ for gaseous CO_2_ and − 4‰ for DIC, confirms the mantellic and volcano-magmatic CO_2_ sources (about − 4‰) at FLFF^7,22^.

At SBHS, ground gas CO_2_ concentration, measured in the fumaroles, gives a high mean value of 95 ± 2%. Thermal waters have more DIC than at FLFF, with 31 ± 1 × 10^−3^ mol L^−1^ on average. Mean δ^13^C of − 0.72 ± 0.01‰ for gaseous CO_2_ and 0.5 ± 0.4‰ for DIC, confirms a metamorphic CO_2_ source (from 0 to − 2‰) at SBHS^46^. Finally, surface and ground gas temperatures are higher at FLFF compared with SBHS (Table 2).

### Radon‒CO_2_ fluxes correlation

**Figure 5:**
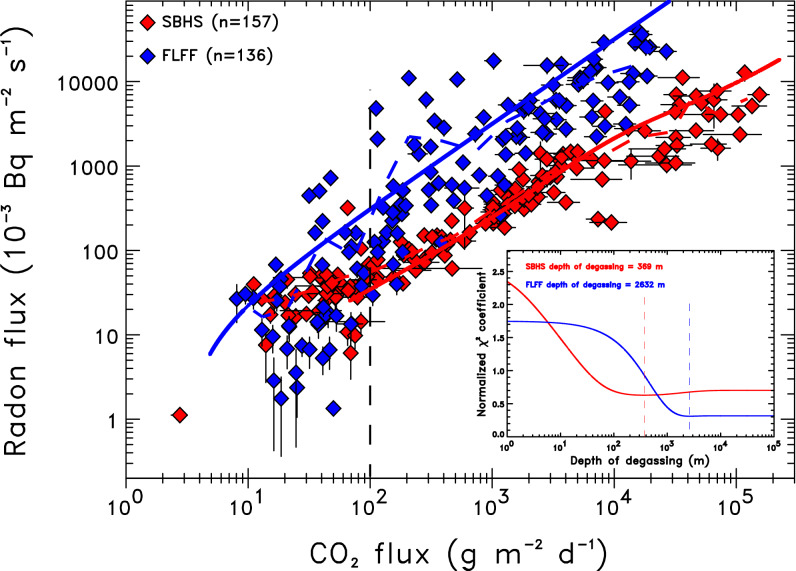
Radon‒CO_2_ fluxes correlation for FLFF (in blue) and SBHS (in red). Only fluxes measured after the 2015 Gorkha earthquake are considered at SBHS. Diamond shows the data-set, dashed curve represents the average of the data, and solid curve is the calculation of the advective–diffusive radon transport model separately for each site using the depth of degassing constrained by an unbiased approach with 500 simulations (see “Methods” section). The bottom right inset represents the normalized *χ*^2^ coefficient as a function of the depth of the radon source, constraining the depth of CO_2_ degassing separately for each site (369 m for SBHS and 2632 m for FLFF). Model parameters are summarised in Table 3.

A general spatial agreement is found between radon flux and CO_2_ flux patterns both at FLFF (Supp. Fig. S2) and SBHS (Supp. Fig. S3). Combined radon and CO_2_ fluxes were measured exactly at the same point at 136 and 157 locations at FLFF and SBHS, respectively (see “Methods” section). High radon‒CO_2_ fluxes correlation coefficients of 0.83 ± 0.02 for FLFF and 0.80 ± 0.03 for SBHS suggest that CO_2_ is the main carrier gas of radon at both sites. Radon and CO_2_ fluxes are indeed strongly correlated over more than five orders of magnitude at both sites (Fig. 5), following a power-law relationship: Φ_Rn_ = 0.974Φ_CO2_^1.053^ (*R*^2^ = 0.78) for FLFF, and Φ_Rn_ = 2.441Φ_CO2_^0.681^ (*R*^2^ = 0.90) for SBHS. Despite a high dispersion of flux values for each site, systematic differences are larger than the dispersion and both sites can be discriminated. For a given CO_2_ flux, larger radon flux is observed at FLFF compared with SBHS, compatible with the larger radon source term of rocks at FLFF. The larger dispersion for FLFF may be explained by the larger range of radon source terms. As radon is carried by CO_2_ to the surface, radon transport mechanism is dominated by diffusion when CO_2_ flux remains small (< 100 g m^−2^ day^−1^), and by advection when CO_2_ flux increases^47,56,58^. For CO_2_ fluxes > 100 g m^−2^ day^−1^ in the advective domain (Fig. 5), radon fluxes are systematically larger at FLFF, and reach particularly high values for CO_2_ flux > 1000 g m^−2^ day^−1^, while, at SBHS, they tend to saturate. This confirms the reliability of our approach and suggests a shallower gas source at SBHS, a concept which is confirmed below by a detailed calculation.

To interpret further Fig. 5, indeed, we consider a simplified model, less complex than the reality of the two sites, but able to reproduce the essence of the radon signature of CO_2_. We use an updated advective–diffusive transport model of radon carried upward by CO_2_ to the surface^58^ (see “Methods” section; Supp. Fig. S6). This model considers three phases for radon (gaseous, dissolved, and adsorbed) and three layers (soil, rock, and deep rock), and was modified to include temperature- and water-saturation-dependence of several radon parameters, including radon source term, an important modification for hydrothermal and volcanic sites. Since most parameters are assessed from field and laboratory data-sets, the model is able to represent both sites in term of radon transport from source(s) to surface. Deep rock layer is assumed saturated and its temperature is set to the vapour/liquid equilibrium temperature. For each layer, fixing porosity, water saturation, gas temperature, and radon source term, the depth of the deepest interface (rock ‒ deep rock interface) can be calculated using published equations^58^ (see “Methods” section; Table 3). We optimise this interface depth using a normalised *χ*^2^ coefficient calculated for each data-set (*i* number of data with CO_2_ flux > 100 g m^−2^ day^−1^), *χ*^2^ = Σ_*i*_(Φ_Rn*i*_^meas^‒Φ_Rn*i*_^calc^)^2^/(σ_ΦRn*i*_^2^ + σ_ΦCO2*i*_^2^), where, respectively, Φ_Rn_^meas^ and Φ_Rn_^calc^ are measured and calculated radon fluxes, and σ_ΦRn_ and σ_ΦCO2_ are one-sigma uncertainty on radon and CO_2_ fluxes. This interface corresponds to the maximum depth of the radon source, where CO_2_ velocity is high enough to carry radon before its decay, and therefore represents the maximum depth of radon-carrier degassed CO_2_. In some cases, it corresponds to the maximum depth of CO_2_ degassing. In other cases, CO_2_ degassing can be deeper and this depth can be seen as a minimum depth for degassed CO_2_.

**Table 3:** Parameters used in the advective–diffusive model of radon transport separately for FFLF and SBHS.

Site	FLFF	SBHS
**Soil layer**		
Thickness (m)	1	1
Porosity (%)	10	25
Water saturation (%)	80	50
Gas temperature (°C)	29.6 (29.6 ± 0.8; 500 simulations)	16.3 (16.3 ± 0.8; 500 simulations)
Water–air partition coefficient	0.20 (recalculated; 500 simulations)	0.29 (recalculated; 500 simulations)
Adsorption coefficient (m^3^ kg^−1^)	4.0 × 10^−5^ (recalculated; 500 simulations)	5.7 × 10^−5^ (recalculated; 500 simulations)
Corrected *EC*_Ra_ (Bq kg^−1^)	6.6 (6.6 ± 0.5; 500 simulations)	8.1 (8.1 ± 0.6; 500 simulations)
**Rock layer**		
Constrained thickness (m)	2632 (2580 ± 180; 500 simulations)	369 (380 ± 20; 500 simulations)
Porosity (%)	5	5
Water saturation (%)	80	50
Gas temperature (°C)	45.8 (45.7 ± 3.0; 500 simulations)	25.8 (25.7 ± 0.9; 500 simulations)
Water–air partition coefficient	0.15 (recalculated; 500 simulations)	0.22 (recalculated; 500 simulations)
Adsorption coefficient (m^3^ kg^−1^)	3.8 × 10^−5^ (recalculated; 500 simulations)	6.3 × 10^−5^ (recalculated; 500 simulations)
Corrected *EC*_Ra_ (Bq kg^−1^)	5.0 (5.0 ± 0.9; 500 simulations)	2.3 (2.3 ± 0.5; 500 simulations)
**Deep rock layer**		
Porosity (%)	5	5
Water saturation (%)	100	100
Gas temperature (°C)	270	120
Water–air partition coefficient	0.1	0.11
Adsorption coefficient (m^3^ kg^−1^)	3.8 × 10^−5^	6.3 × 10^−5^
Corrected *EC*_Ra_ (Bq kg^−1^)	22	0.58

The thickness of the rock layer, constrained by 500 simulations, gives an estimate of the depth of degassing (see text).

Gas temperature and radon source term having larger effects on degassing depth (“Methods” section; Supp. Fig. S7), we vary these parameters of the soil and rock layers around the mean with 500 simulations. Minimum normalised *χ*^2^ coefficients give an optimized depth of degassing for each simulation, and the median of their distribution (Supp. Fig. S8) yields 2580 ± 180 m for FLFF and 380 ± 20 m for SBHS (Table 3 and inset of Fig. 5). Injecting these constrained depths, the model reproduces well the radon‒CO_2_ fluxes correlation over four to five orders of magnitude (Fig. 5). Furthermore, these depths match the general overview of CO_2_ transport at both sites, as described above. At FLFF, degassing depth of 2.6 km is compatible with the presence of a crystallised body of mica-rich syenite, which previous studies attributed a depth of 3‒4 km^23^. At SBHS, degassing depth of 380 m is consistent with a shallow CO_2_ reservoir, sensitive to crustal deformation and earthquakes^8^. While the interpretation of the estimated depth of degassing, when it is large, needs to be cautious, the one-order-of-magnitude difference in degassing depth between FLFF and SBHS is evidenced without doubt. This difference could also be examined through the prism of the CO_2_ source, as volcanic settings with mantellic and volcano-magmatic CO_2_ might have deeper roots than collisional settings with metamorphic CO_2_.

## Discussion

The results shown above indicate that the most likely source of radon is from the rock. However, other radon sources have been considered: the thermal waters and the soil. At both sites, a water-degassing model^58^ (Supp. Fig. S9; see “Methods” section) or a purely diffusive model^58^ (Supp. Fig. S10; see “Methods” section) cannot account for the obtained estimated radon and CO_2_ discharges or reproduce the whole data-set, respectively. Thus, the rock layer appears the most representative radon source, controlling radon production and whose thickness constrains the degassing depth at both sites. The degassing depth appears nevertheless better constrained at SBHS, where depths greater than a few hundreds of metres are unlikely, whereas at FLFF, depths of ≈ 10 km might be possible (Supp. Figs. S8 and S11). Our model clearly interprets the unambiguous observation of the saturation of the radon flux at high CO_2_ flux as a smaller source thickness. At high CO_2_ velocities, the path length in the rock is not sufficient to recharge the CO_2_ flow with radon.

Our model takes into account the temperature dependence of radon source term and other radon parameters, but only experimentally constrained in the range 0‒100 °C (see “Methods” section), highlighting also limitations of our approach. Too little information on radon parameters variation with higher temperature is available^64^, motivating future investigations in that direction. Similarly, at such degassing depths (hundreds to thousands of metres), confining pressure is high, but, despite few studies on granitic^13^ and volcanic rocks^12^, radon source term variation with increasing pressure remains poorly known. Nevertheless, our model indicates that the variations of the source parameters or their heterogeneity are second-order effects. The largely dominating effect remains in all reasonable instances the path length, and hence the source depth.

The combination of field measurements of coupled CO_2_ and radon fluxes at the surface, laboratory characterisation of radon source term, and radon transport modelling have constrained the depth of CO_2_ degassing at two hydrothermal sites in different tectonic contexts and with different CO_2_ sources. Our results thus attest to the relevance of gas flux monitoring, particularly important at sites under near-critical conditions, sensitive to Earth’s deformation and earthquakes, which, contrary to what has been done in the past^35,65^, are not exclusively found in volcanic regions. For example, the *M*_w_7.9 2015 Gorkha earthquake greatly affected gas emissions at SBHS, likely liberating CO_2_ previously stored in a crustal reservoir at shallow depths^8^. Our combined approach shows that emitted radon may have changed from a deeper (≈ 1000 m) to a shallower source (≈ 100 m) following the earthquake (Supp. Fig. S12).

Our combined study has revealed to be a powerful tool to determine the depth of degassing at FLFF, with mantellic and volcano-magmatic CO_2_ sources, and at SBHS, with a metamorphic CO_2_ source. To date, only few data are reported on CO_2_ and radon fluxes together^47^, almost all of them in collision context. Our approach should be systematically applied to sites in other tectonic contexts, such as rifting, reverse fault, strike-slip fault, or subduction, as well as in other volcanic environments. In addition, our model was found particularly sensitive for CO_2_ flux > 5000 g m^−2^ day^−1^, motivating future improvement in the measurement of such high fluxes. The presence of high gas fluxes, especially CO_2_, will be important to investigate in more details in the future, in particular when re-evaluating global carbon budgets^9,66,67^.

The radioactive gas radon, tracking CO_2_ degassing and diagnosing CO_2_ transport mechanisms, emerges as a powerful asset to characterise gaseous emissions and monitor earthquake-sensitive geosystems. In addition, the exceptionally high radon discharge from FLFF (≈ 9 GBq day^−1^), during a quiescent period, raises the issue of unconstrained radon emission from volcanoes, and suggests significant radon output from volcanic areas worldwide, especially when a large eruption occurs. Because emissions from volcanoes, unlike background diffusive soil emission, can reach above the atmospheric boundary layer, radon release from volcanoes worldwide may have substantial effects on atmosphere ionisation, aerosol formation, and climate^68^.

## Methods

### Radon flux

Radon-222 flux was measured at the surface using the accumulation chamber method^46,56^. Increase rate with time of radon activity concentration inside the chamber, directly related to radon flux, was measured using scintillation flasks (Algade, France) at both sites. Radon concentration in the flasks was inferred 3.5 h after sampling from counting in photomultipliers (CALEN™, Algade, France), regularly inter-calibrated in the laboratory. The method is robust, even in remote location^48^, and reliable where radon fluxes range over several orders of magnitude^54^. Radon flux is expressed in 10^−3^ Bq m^−2^ s^−1^. Associated uncertainty (Supp. Fig. S1a) was estimated from several systematic tests^54^. Relative experimental uncertainty ranges from 15% for fluxes ≈ 100 × 10^−3^ Bq m^−2^ s^−1^ to 30% for fluxes ≈ 10 × 10^−3^ and ≈ 10,000 × 10^−3^ Bq m^−2^ s^−1^. Radon fluxes were measured during stable weather conditions, in summer 2016 at FLFF, and in winters 2009‒2011, 2015, and 2016 at SBHS. A majority of points were measured several times (from 2 to 18 times); point-averages are arithmetic means. At FLFF, based on several measurements along time at selected points, variations of radon flux were < 10% and < 15% for fluxes < 1000 × 10^−3^ and > 10,000 × 10^−3^ Bq m^−2^ s^−1^, respectively. At SBHS, temporal variations of radon flux were generally < 30% for all fluxes. Total radon discharge, expressed in MBq day^−1^ (or Bq s^−1^), was estimated following the sequential Gaussian simulations (sGs) method^69^ with 100 equiprobable realizations. Radon fluxes measured at SBHS (gas zones 1 and 2) were published^8,47,54^ (Table 1).

### CO_2_ flux

CO_2_ flux was measured at the surface using the accumulation chamber method^70^. Increase rate with time of CO_2_ concentration inside the chamber, directly related to CO_2_ flux, was measured by several portable sensors: at FLFF, two portable infrared fluxmeters (WestSystem™ CO_2_Flux, Italy), regularly calibrated by the manufacturer and inter-calibrated in the field; at SBHS, two portable infrared CO_2_ sensors and home-made accumulation chambers (Testo™ 535, Testo AG, Germany; Vaisala™ CARBOCAP® Hand-Held GM70, Finland), regularly inter-calibrated in the laboratory. Portable instruments are robust, even in remote location^48^ or during monsoon^56^, and reliable where CO_2_ fluxes range over six orders of magnitude^32,54^. CO_2_ flux is expressed in g m^−2^ day^−1^. Associated uncertainty (Supp. Fig. S1b) was estimated from previous assessments^71^ and systematic tests^54^. Relative experimental uncertainty ranges from 10% for low fluxes (10 g m^−2^ day^−1^) to 30% for large fluxes (10,000 g m^−2^ day^−1^). CO_2_ fluxes were measured during stable weather conditions, in summer 2016 at FLFF, and in winters 2015 to 2020 at SBHS. Every point was measured several times (from 2 to 26 times); point-averages are arithmetic means. At FLFF, only 4.3% temporal variation of CO_2_ flux was recorded at the 1-h continuous fluxmeter station GFUR2 during the 8-day-long field campaign (mean: 268 ± 12 g m^−2^ day^−1^). At SBHS, several points measured along time during the numerous field campaigns from November 2015 to January 2020 showed variations < 10% for fluxes < 100 g m^−2^ day^−1^ and < 20% for fluxes > 1000 g m^−2^ day^−1^. Total CO_2_ discharge, expressed in t day^−1^ (or mol s^−1^), was estimated following the sGs method^69^ with 100 equiprobable realizations. CO_2_ fluxes measured from November 2015 to January 2018 at SBHS (gas zones 1 and 2) were published^8^ (Table1).

### Radon‒CO_2_ fluxes correlation

Only values of radon and CO_2_ fluxes obtained at the same measurement points were considered and no interpolation was used. This represents a total of 136 and 157 locations of combined fluxes for FLFF and SBHS, respectively.

### Radon sources

#### Effective radium-226 concentration (EC_Ra_)

Radon-222 source in porous materials is the effective radium-226 concentration, expressed in Bq kg^−1^, i.e. the product of the bulk radium concentration *C*_Ra_ and the emanation coefficient *E*, probability that a radium atom produces a radon atom in the pore space. *EC*_Ra_ was measured in the laboratory on rock and soil samples using a radon accumulation method^60,72,73^. The sample was placed in a hermetically closed container. After a 5-to-18-day accumulation time, radon concentration of the free air inside the accumulator was determined after sampling using a scintillation flask (Algade, France) and counting in a photomultiplier (CALEN™, Algade, France). A minimum of three measured values per sample were averaged. Relative experimental uncertainty ranges from 3% for *EC*_Ra_ ≈ 30 Bq kg^−1^ to 10% for *EC*_Ra_ ≈ 1 Bq kg^−1^. SBHS data were partly published^50,54^.

#### Ground radon concentration

Ground radon-222 concentration, expressed in Bq m^−3^, was measured at FLFF at 60‒100 cm depth by gas pumping using a portable RAD7 detector (Durridge Company, Inc., USA). At SBHS, it was measured at 100 cm depth using continuous radon concentration probes (Barasol™ and BMC2™, Algade, France). All these instruments are based on the detection of alpha particles by a silicon detector material. Sensitivity, inter-comparison dispersion, and overall common uncertainty are 4 and 50 Bq m^−3^, 5% and 3%, and 5% and 5%, for RAD7 and continuous instruments, respectively. SBHS data were partly published^54,57^.

#### Radon in water bubbles

Radon-222 concentration in water bubbles was measured after accumulation of gas in small containers above the water pond, sampling using scintillation flasks, and counting in photomultipliers. Relative experimental uncertainty is similar to that of *EC*_Ra_ method.

#### Radon and radium in water

Radon-222 concentration in water, expressed in Bq L^−1^, was measured by the emanometry method^58,62^. The water was sampled in a container, hermetically closed after sampling. After shaking, sampling of the air inside the container was done by a scintillation flask and counting using a photomultiplier. Relative experimental uncertainty ranges from 5 to 30%. Two or more replicates were performed at each location. Radium-226 concentration in water, expressed in 10^−3^ Bq L^−1^, was measured following the radon method^58,62,74^. After water sampling in the field, the container was kept closed in the laboratory for 2 months minimum before measurement. Relative experimental uncertainty ranges from 5% for *C*_Ra_ ≈ 100 × 10^−3^ Bq L^−1^ to 10% for *C*_Ra_ ≈ 10 × 10^−3^ Bq L^−1^ (Table 2).

### CO_2_ sources

#### Ground CO_2_ concentration

Ground CO_2_ concentration, expressed in vol.%, was measured at FLFF at 60‒100 cm depth by gas pumping using a portable infrared sensor (Geotechnical Instruments, UK) with 0‒100 vol% measurement range. At SBHS, gas was sampled using evacuated glass tubes and CO_2_ concentration was determined manometrically^54^. Absolute experimental uncertainty ranges from 0.5 to 3% for the infrared cell and from 0.1 to 1% using the mass spectrometer. Only 2007‒2018 SBHS data were partly published^8,46,47,54^.

#### δ^13^C of CO_2_ gas

The gas was collected using accumulation chambers and evacuated glass tubes^8,47,54^. Gas samples were analysed for molecular composition in the laboratory^45^. Carbon isotopic ratio of gaseous CO_2_, δ^13^C, expressed in ‰ and defined relative to the standard value of Pee Dee belemnite (V-PDB), was determined after off-line purification using a Finnigan™ MAT-253 mass spectrometer (Thermo Electron Corp., Germany) at CRPG (Nancy, France). Repeatability and absolute experimental uncertainty are 0.1 ‰. FLFF data were partly published^7,22^; SBHS 2007‒2018 data were published^8,46,47,54^.

#### Dissolved inorganic carbon concentration and δ^13^C of dissolved CO_2_

Water was sampled using 12-mL screw cap vials. Dissolved inorganic carbon (DIC) concentration, expressed in 10^−3^ mol L^−1^, was determined using a gas chromatograph coupled to an isotope ratio mass spectrometer (GCIRMS, GV 2003, GV Instruments, UK) at IPGP (Paris, France). Relative experimental uncertainty ranges from 1 to 2%. δ^13^C of dissolved CO_2_ in water, expressed in ‰ relative to V-PDB, was determined using the same spectrometer at IPGP. Absolute experimental uncertainty is 0.1‰. SBHS data were published^8^; FLFF data were taken from the literature^24^ (Table 2).

### Surface and ground temperature

#### Surface temperature

At FLFF, temperature was measured systematically at surface using a thermocouple (Testo 925™, Testo AG, Germany). Absolute experimental uncertainty is 0.1 °C. At SBHS, temperature was measured at the surface using regularly inter-calibrated, autonomous sensors (SB39, Seabird™, USA). Their sensitivity is better than 3.5 × 10^−3^ °C^54^.

#### Ground temperature

At FLFF, temperature was measured at the top of 60‒100 cm holes with the same thermocouple used for surface temperature. Absolute experimental uncertainty is 0.1 °C. At SBHS, temperature was measured at 100 cm depth using SB39 sensors, recording at 30 s intervals (Table 2).

### Radon transport modelling

#### Diffusive–advective radon transport model

The model considers that degassing of hydrothermal CO_2_ is initiated at depth, provided it travels fast enough relative to the radon-222 half-life. After degassing, CO_2_ percolates through rock and soil layers until it reaches the surface, carrying radon produced by these layers along its pathway. We consider soil, rock, and deep rock layers from surface in a semi-infinite porous medium (Supp. Fig. S6). For radon, we consider gaseous, dissolved, and adsorbed phases with equation terms of diffusion, advection, radioactive decay, and production, that are implemented analytically. We use transport Eqs. (B2)–(B4) of Ref.^58^ without modification. Using Eqs. (B6)–(B11) or Eqs. (B13)–(B17) for large advection (Ref.^58^), we solve the following steady-state 1D equation for each layer *i*:

$${D}_{\mathrm{S}i}\frac{{\partial }^{2}{C}_{\mathrm{a}i}}{\partial {z}^{2}}-{T}_{\mathrm{S}i}\frac{\partial {C}_{\mathrm{a}i}}{\partial \mathrm{z}}+\uplambda ({C}_{\mathrm{a}i}^{0}-{C}_{\mathrm{a}i})=0,$$

where ${D}_{\textrm{S}i}=\frac{{\upvarepsilon }_{\mathrm{a}i}{D}_{\mathrm{e}i}^{\mathrm{a}}+{\varepsilon }_{\mathrm{w}i}{\kappa }_{\mathrm{w}i}{D}_{\mathrm{e}i}^{\mathrm{w}}}{{\upvarepsilon }_{\mathrm{a}i}+{\varepsilon }_{\mathrm{w}i}{\kappa }_{\mathrm{w}i}+{k}_{{\text{d}}i}\rho }$, ${T}_{\mathrm{S}i}=\frac{u+I{\kappa }_{\mathrm{w}i}}{{\upvarepsilon }_{\mathrm{a}i}+{\varepsilon }_{\mathrm{w}i}{\kappa }_{\mathrm{w}i}+{k}_{{\text{d}}i}\rho }$ and equilibrium radon concentration ${C}_{\mathrm{a}i}^{0}=\frac{{EC}_{{\text{Ra}}i}\uprho }{{\upvarepsilon }_{\mathrm{a}i}+{\varepsilon }_{\mathrm{w}i}{\kappa }_{\mathrm{w}i}+{k}_{{\text{d}}i}\rho }$, with *u* the Darcy velocity related to the carrier CO_2_ flux and *I* the water infiltration (for more details see Supp. Fig. S6 and Ref.^58^). Here, for given layer i, we introduce empirical relations for porosity- and water-saturation-dependent effective diffusion coefficients^75^ (${D}_{\mathrm{e}i}^{\mathrm{a}}=1.1\times {10}^{-5}\times {\upvarepsilon }_{\mathrm{a}i}\mathrm{exp}(-6{S}_{\mathrm{w}i}{\varepsilon }_{\mathrm{a}i}-6{{S}_{\mathrm{w}i}}^{14 {\upvarepsilon }_{\mathrm{a}i}})$ and ${D}_{\mathrm{e}i}^{\mathrm{w}}=1.1\times {10}^{-8}\times {\upvarepsilon }_{\mathrm{w}i}\mathrm{exp}(-6\left(1-{S}_{\mathrm{w}i}\right){\varepsilon }_{\mathrm{w}i}-6(1-{{S}_{\mathrm{w}i})}^{14 {\upvarepsilon }_{\mathrm{w}i}})$), temperature-dependent water/air partition^58^ and adsorption coefficients^76^ (${\kappa }_{{\text{w}}i}=0.104+0.416\mathrm{exp}\left(- 0.0491{T}_{i}\right)$ and ${k}_{{\text{d}}i}={k}_{\mathrm{d}i}^{0}\mathrm{exp}\left(\frac{19500}{8.314}(\frac{1}{{T}_{i}}-\frac{1}{253.15})\right)$ with ${k}_{\mathrm{d}1}^{0}=0.181$ and ${k}_{\mathrm{d2,3}}^{0}=0.258$) and water-saturation- and temperature-dependent radon source terms^73,77^ (${EC}_{{\text{Ra}}1}={EC}_{\mathrm{Ra}1}^{0}(32.22{\text{exp}}\left(-1.88{S}_{\mathrm{w}1}\right)-31.43{\text{exp}}\left(- 1.98{S}_{\mathrm{w}1}\right))(1+0.01(0.7875{T}_{1}-15.75))$) and ${EC}_{{\text{Ra}}\mathrm{2,3}}={EC}_{\mathrm{Ra2,3}}^{0}(32.22{\text{exp}}\left(- 1.88{S}_{\mathrm{w}1}\right)-31.43{\text{exp}}\left(- 1.98{S}_{\mathrm{w}1}\right))(1+0.01(0.5535{T}_{1}-11.07))$). For both sites, we fix parameters according to field and laboratory data-sets, such as soil thickness (1 m), soil and rock porosity and water saturation (deep rock is assumed saturated), gas temperature, and radon source term; we calculate water–air partition and adsorption coefficients; and we correct radon source term. For a given CO_2_ flux, we calculate a radon flux using Eqs. (B12) or (B18) for large advection (Ref.^58^). We then compare the calculated and measured radon fluxes using a χ^2^ coefficient (see text) to estimate the best depth of degassing (interface between the rock and deep rock layers). We separately tested the sensitivity of the degassing depth to several parameters considering an acceptable range of values (Supp. Fig. S7). For the soil and deep rock layers, varying total porosity (soil: 0.01‒0.4, rock: 0.01‒0.15), water saturation (0.1‒0.9), gas temperature and radon source term (50% around the mean; see Table 2) has low effect on the degassing depth. However, such variations for the rock layer yield larger effects (Supp. Fig. S7), in particular for gas temperature and radon source term. Then, to explore the phase space, similarly to a Bayesian Monte Carlo unbiased approach, we generated 500 independent models, varying gas temperature and radon source term in the soil and rock layers, with Gaussian distributions, to estimate the depth of degassing (see text; Table 3). The final degassing depth estimate is taken as the median value from the 500 simulations (Supp. Fig. S8).

#### Alternative models

Other modelling approaches were also used. A first analytical model^58^ considers that a given fraction *f* of CO_2_ degasses together with radon from water springs at or near the surface. We use Eqs. (A1)–(A7) of Ref.^58^ without modification. Another analytical model^58^ considers that radon transport is only governed by diffusion, using the same equations given above with *u* = 0. We use Appendix C of Ref.^58^. For these two alternative models, the full description and the equations are given in a previous contribution^58^.

## Supplementary Information

Supplementary Figures.

## Data Availability

The datasets generated and analysed during the current study are available from the corresponding author on reasonable request.
