# Supplementary material for: Radon signature of CO2 flux constrains the depth of degassing: Furnas volcano (Azores, Portugal) versus Syabru-Bensi (Nepal Himalayas)
Source: Sci Rep. 2022 Jun 27;12:10837. doi: 10.1038/s41598-022-14653-5 (PMC9237126; doi:10.1038/s41598-022-14653-5)
Supplement: Supplementary file 1 — Supplementary Figures. [file 41598_2022_14653_MOESM1_ESM.pdf]

SUPPLEMENTARY INFORMATION

Radon signature of CO_2 flux constrains the depth of degassing: Furnas volcano (Azores, Portugal) versus Syabru-Bensi (Nepal Himalayas)

by Girault et al.

Supplementary Figures:

Figures S1 – S12

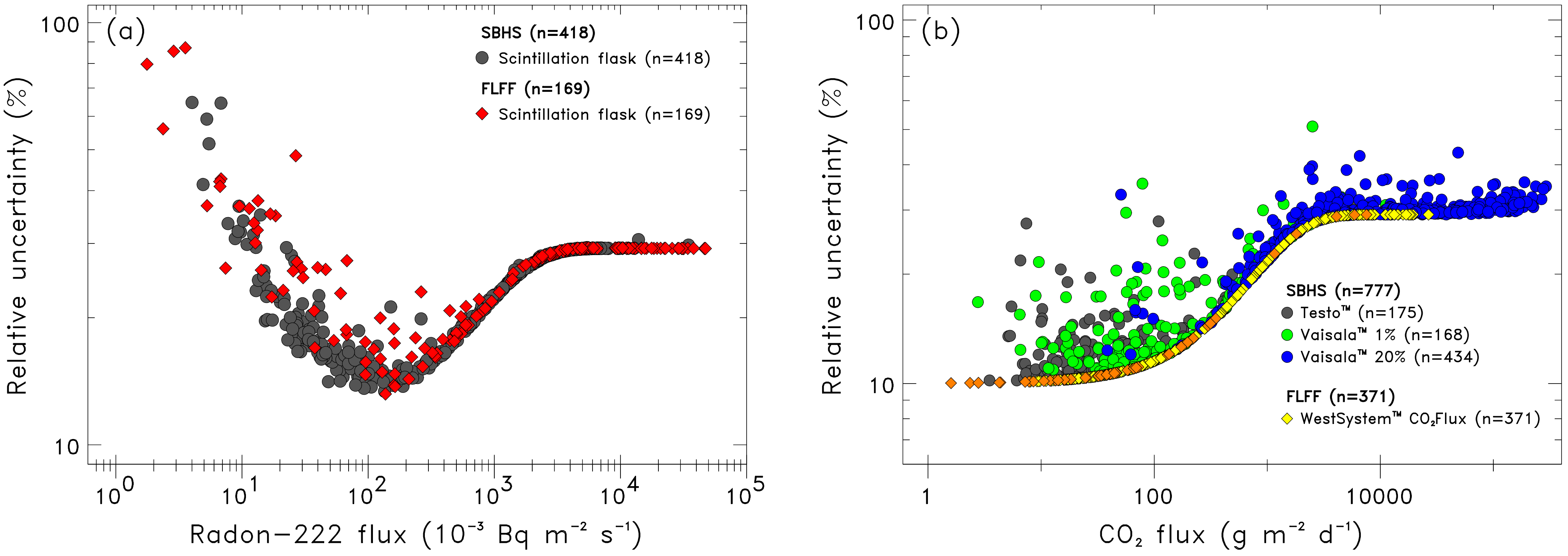

Figure S1. Relative experimental uncertainty of **(a)** radon flux and **(b)** CO_2 flux for both sites. In (a), the relative experimental uncertainty of the use of the scintillation flask method to measure radon flux is shown. In (b), the relative experimental uncertainty associated with the various instruments used to measure CO_2 flux (Testo™, Vaisala™, and WestSystem™ sensors) are shown separately.

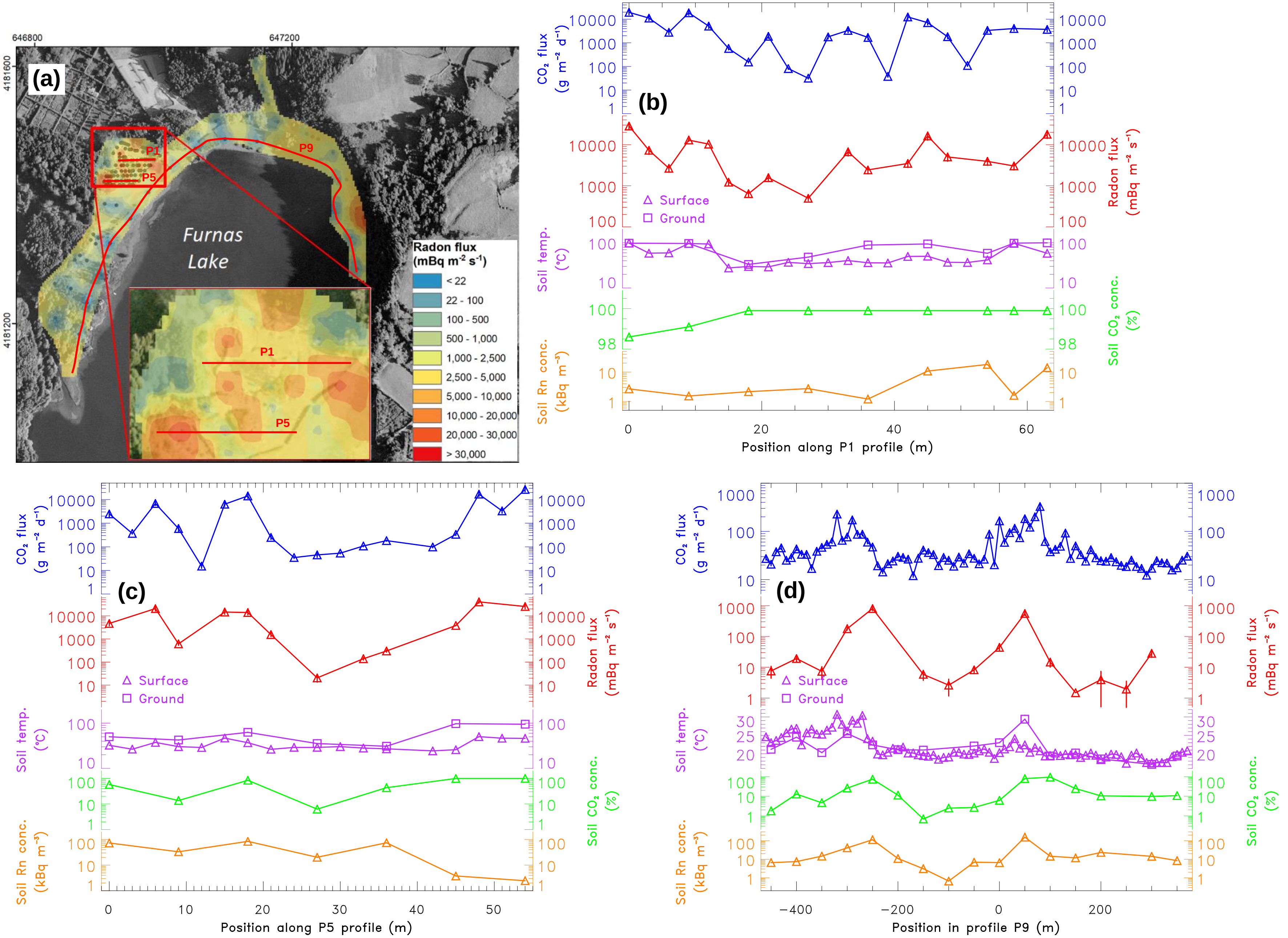

Figure S2. Selected gas flux profiles carried out at FLFF. In (a), the map of FLFF shows the location of the P1, P5, and P9 profiles. In (b, c, and d) are shown as a function of the position along P1, P5 and P9 profiles, respectively, from top to bottom, CO_2 flux (blue), radon flux (red), surface and ground temperature (purple), soil CO_2 concentration (green), and soil radon concentration (orange).

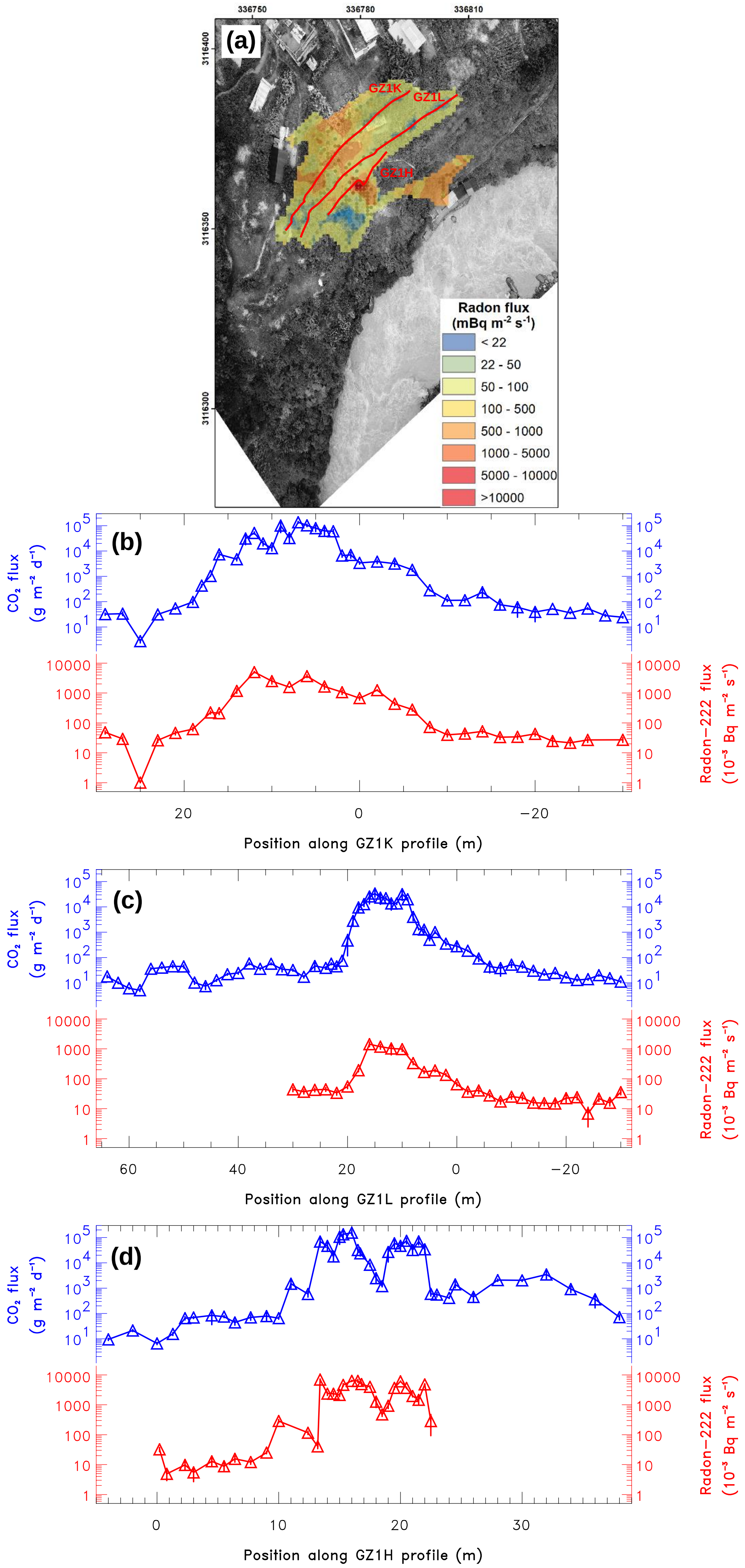

Figure S3. Selected gas flux profiles carried out at SBHS. In (a), the map of SBHS shows the location of K, L, and H profiles. In (b, c, and d) are shown as a function of the position along K, L, and H profiles, respectively, from top to bottom, CO_2 flux (in blue) and radon flux (in red).

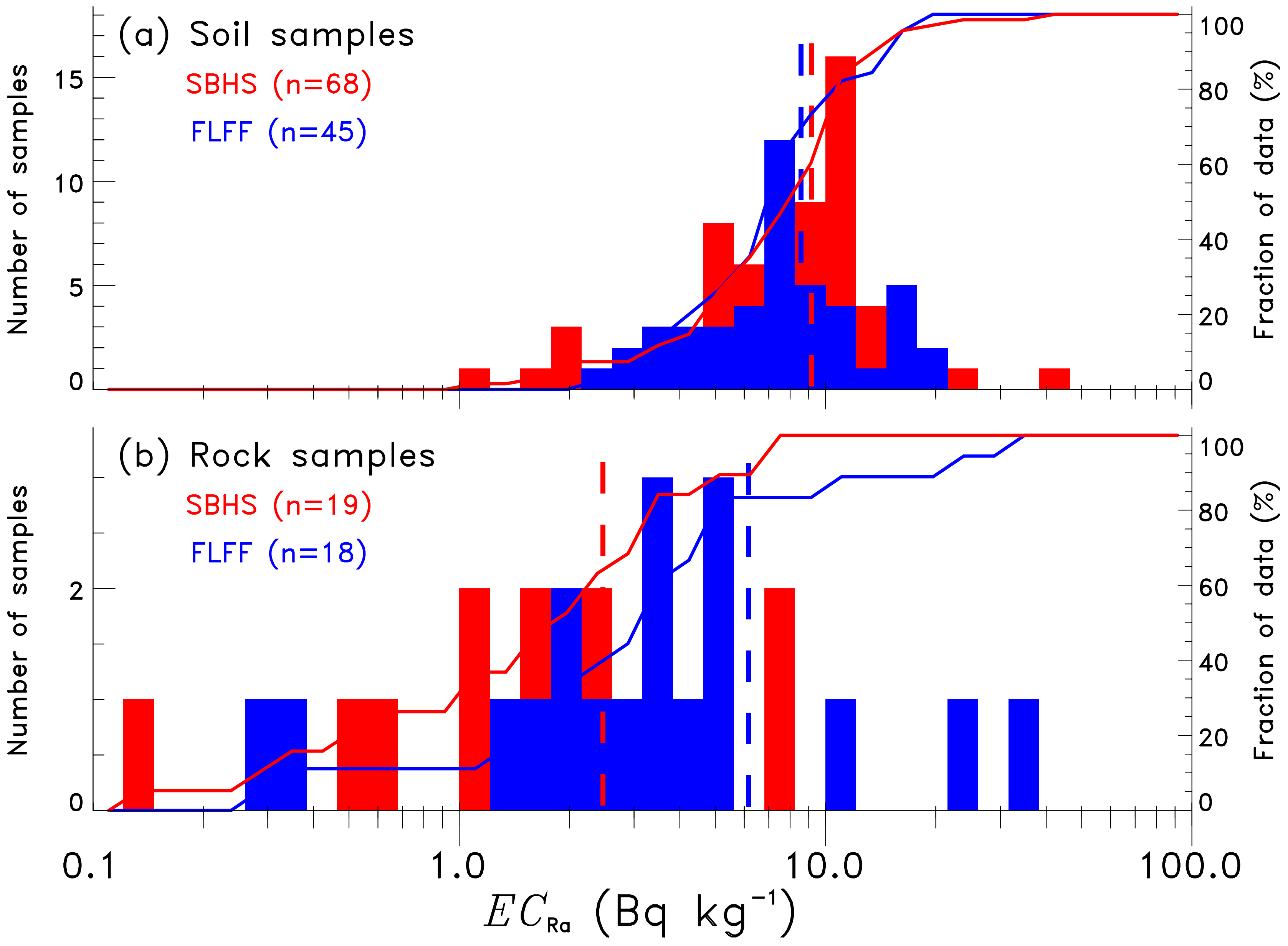

Figure S4. Distributions of effective radium-226 concentrations (EC_{Ra}, radon source term) of **(a)** top soils and **(b)** rocks at FLFF (in blue) and SBHS (in red). Statistics are summarized in Table 2. Cumulated distributions (scale on the right-hand side) are shown as solid curves and arithmetic mean values as vertical dashed lines.

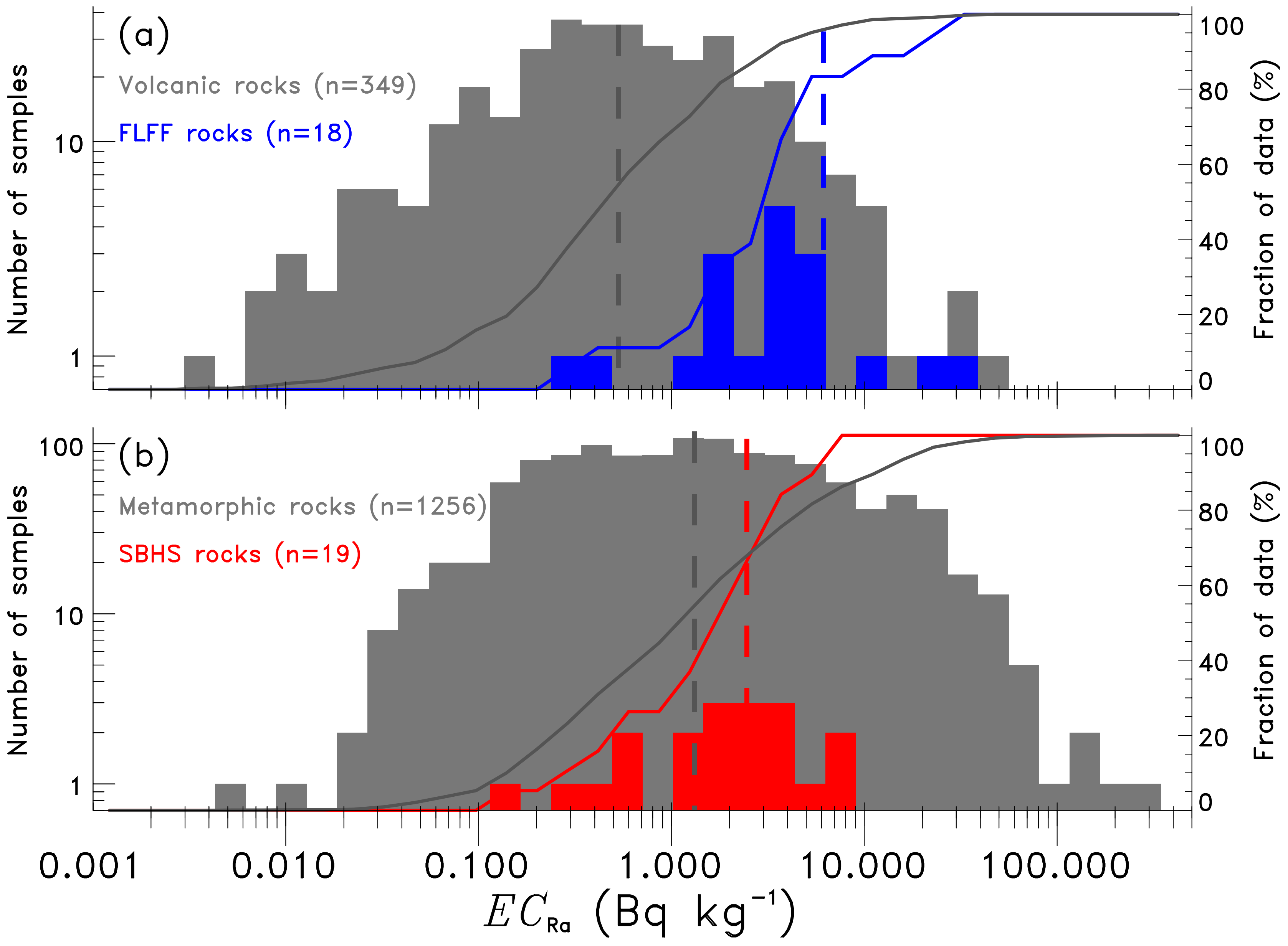

Figure S5. Distributions of effective radium-226 concentrations (EC_{Ra}, radon source term) of **(a)** volcanic rocks (including FLFF rocks in blue) and **(b)** metamorphic rocks (including SBHS rocks in red). Statistics are summarized in Table 2 and given in the text. Cumulated distributions (scale on the right-hand side) are shown as solid curves and arithmetic mean values as vertical dashed lines.

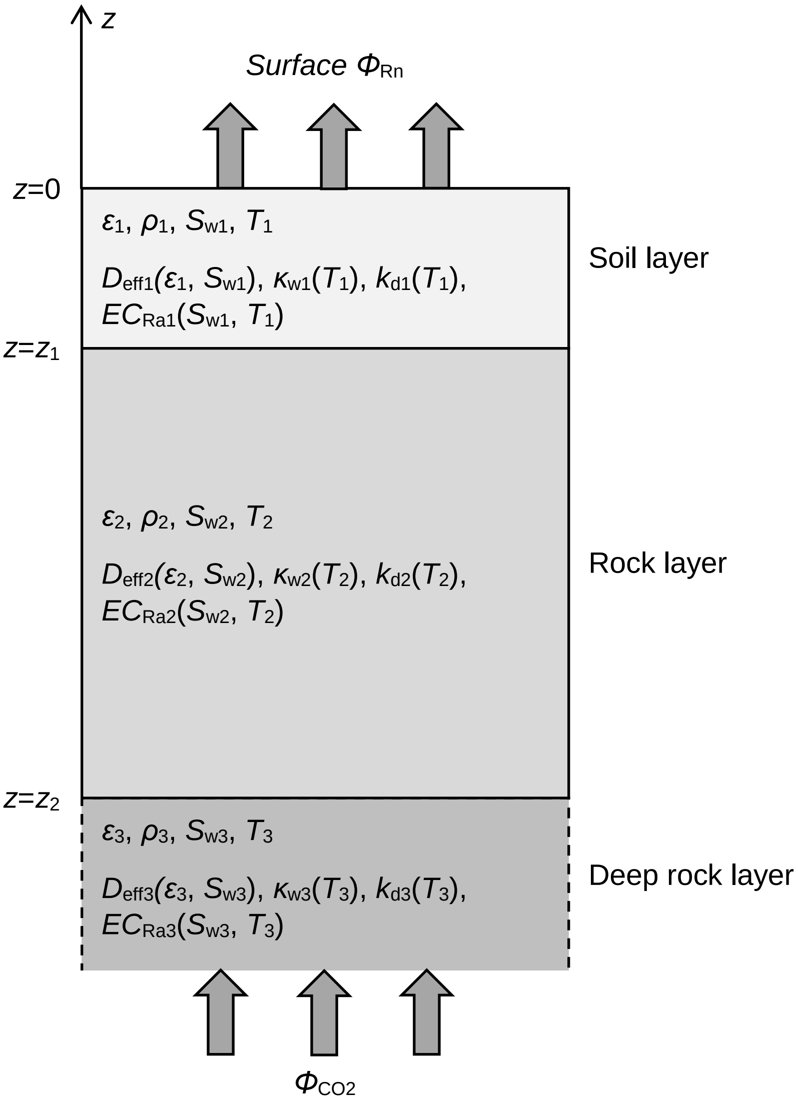

Figure S6. Sketch of the advective-diffusive model of radon transport showing the three layers and their associated physical parameters (see Methods). The symbols ε, ρ, S_w, and T stand for connected porosity, rock density, water saturation, and temperature, respectively. The notations $D_{eff}(\varepsilon, S_w)$, $\kappa_w(T)$, $k_d(T)$, and $EC_{Ra}(S_w, T)$ correspond to porosity- and water-saturation-dependent effective diffusion coefficients, temperature-dependent water/air partition and adsorption coefficients, and water-saturation- and temperature-dependent radon source terms, respectively, as defined in the Methods section.

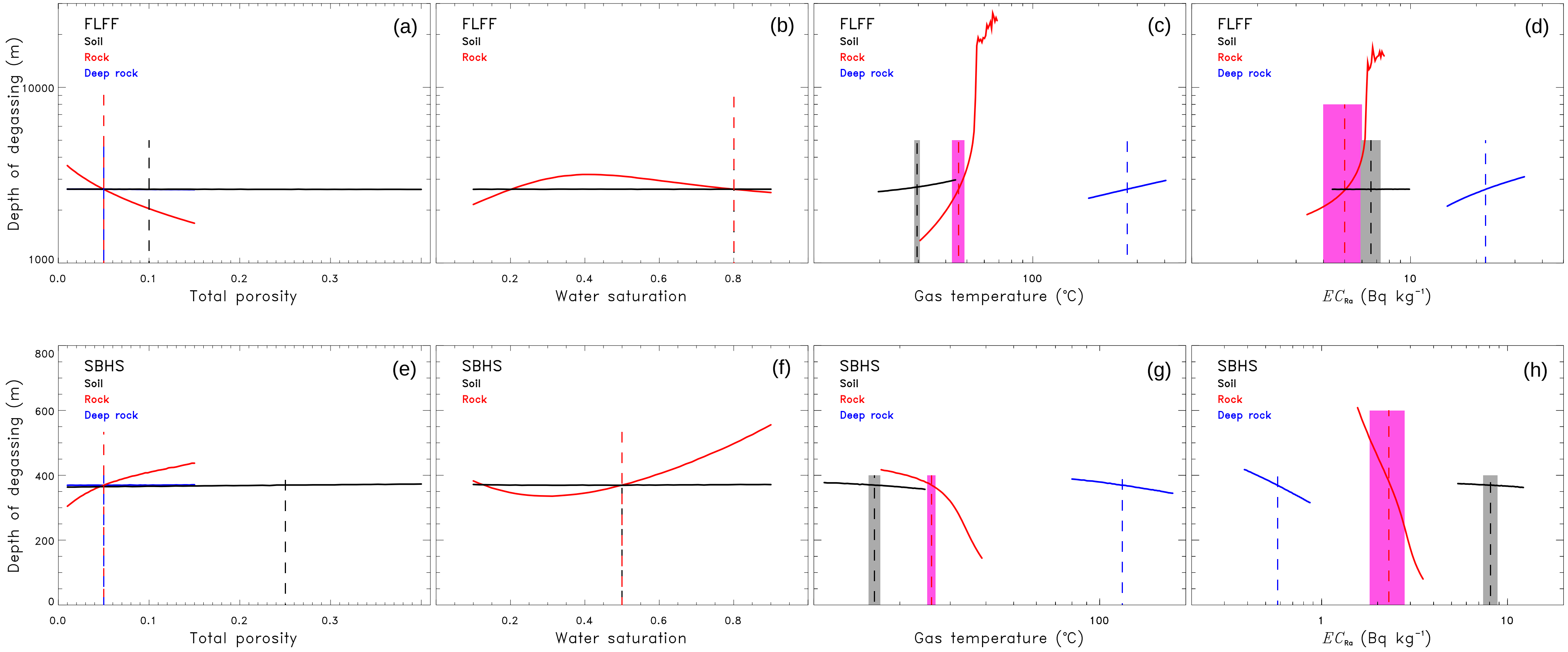

Figure S7. Sensitivity analysis of several parameters in the three layers for the determination of the depth of degassing using the advective-diffusive radon transport model. For FLFF, (a) porosity, water saturation (b), gas temperature (c) and radon source term (d) are varied. For SBHS, (e) porosity, water saturation (f), gas temperature (g) and radon source term (h) are also varied. For each subplot and each layer, the vertical dashed lines represent the value constrained by the available data and the coloured-band the range of variation considered for the simulations whose results are shown in Supp. Fig. S8.

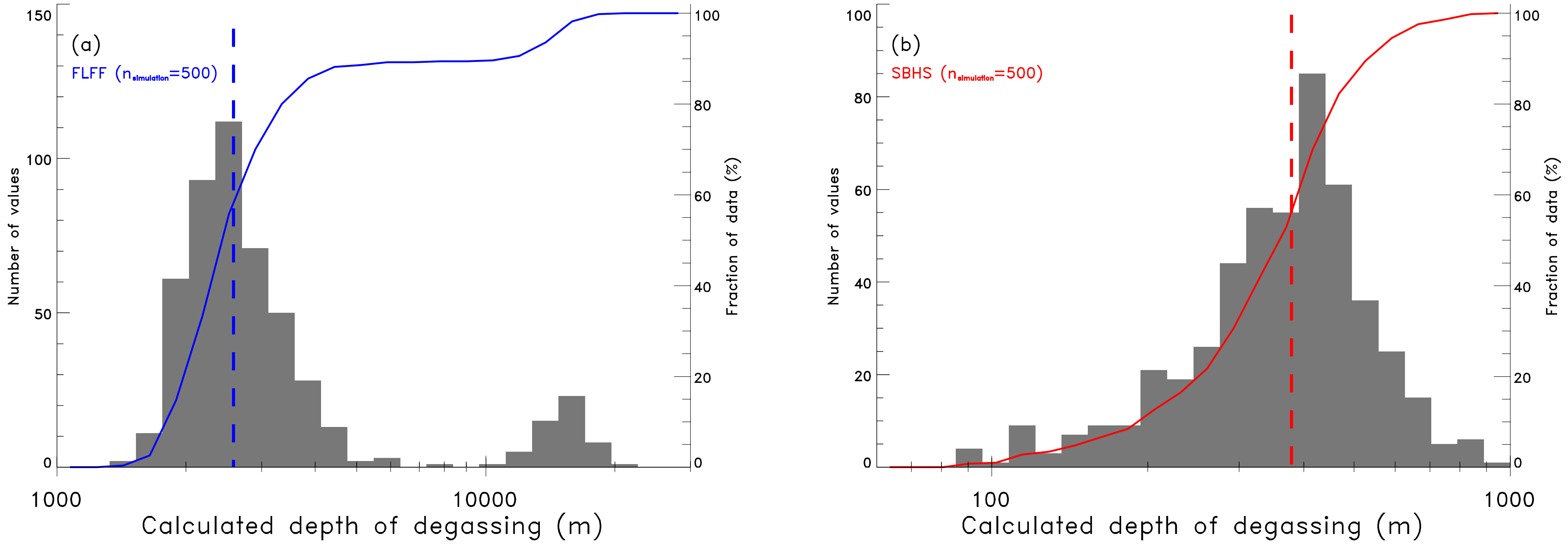

Figure S8. Distribution of the calculated depth of degassing based on 500 simulations for (a) FLFF and (b) SBHS. Gas temperature and radon source term are varied around the mean. Cumulated distributions (scale on the right-hand side) are shown as solid curves and median values as vertical dashed lines.

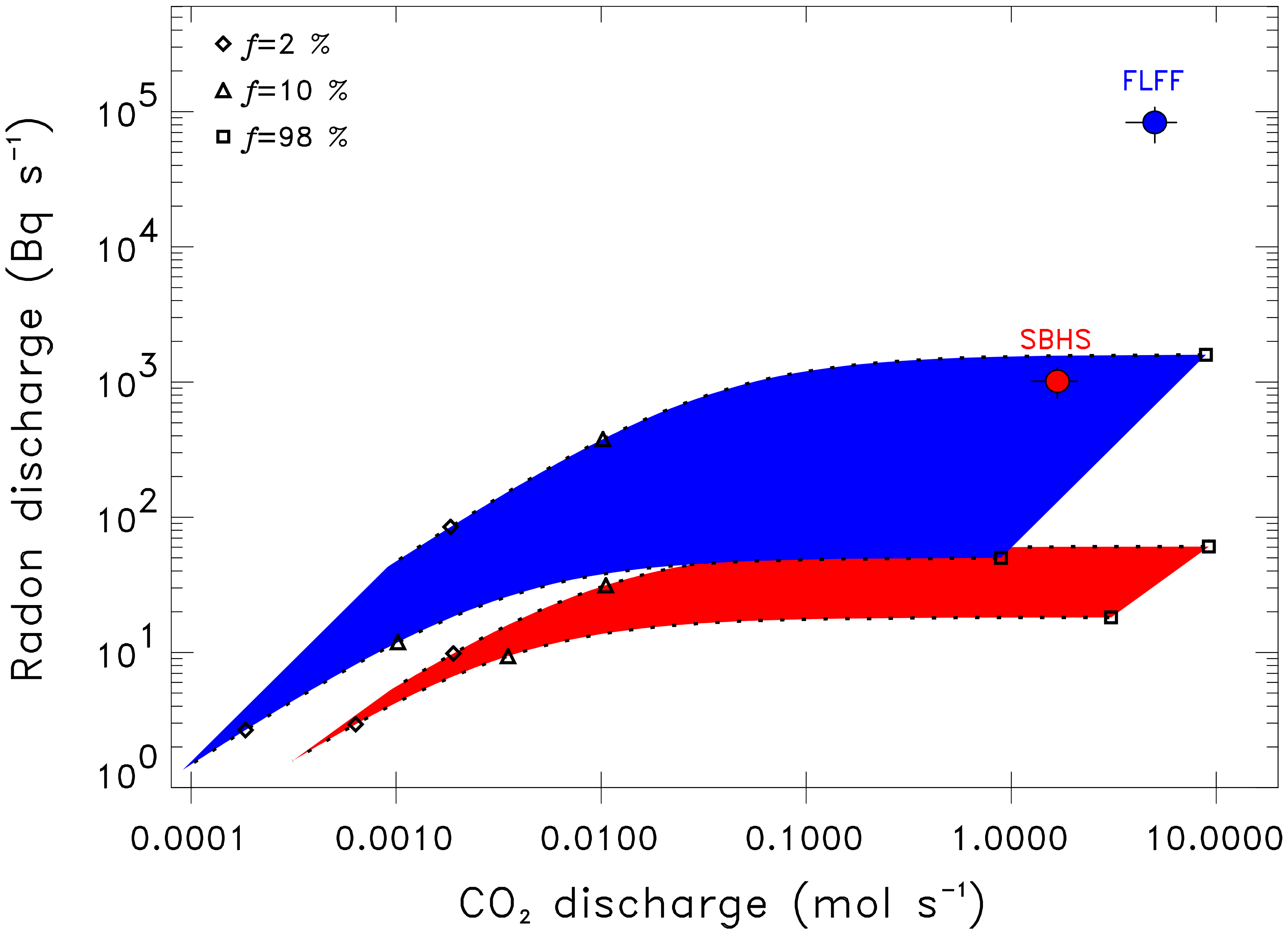

Figure S9. Radon discharge as a function of CO_2 discharge for both sites from the water degassing model. Estimated discharges are shown as circles (in blue for FLFF and in red for SBHS). Model calculations are shown as coloured bands accounting for the range of values of data and model parameters: for FLFF, 0.1 m ≤ fault zone width ≤ 1 m, 6.2 ≤ EC_{Ra} of the deep zone ≤ 20 Bq kg^{-1}, and 1 ≤ water flowrate ≤ 10 L s^{-1}; for SBHS, 0.01 m ≤ fault zone width ≤ 1 m, 0.4 ≤ EC_{Ra} of the deep zone ≤ 2.5 Bq kg^{-1}, and 1 ≤ water flowrate ≤ 3 L s^{-1}.

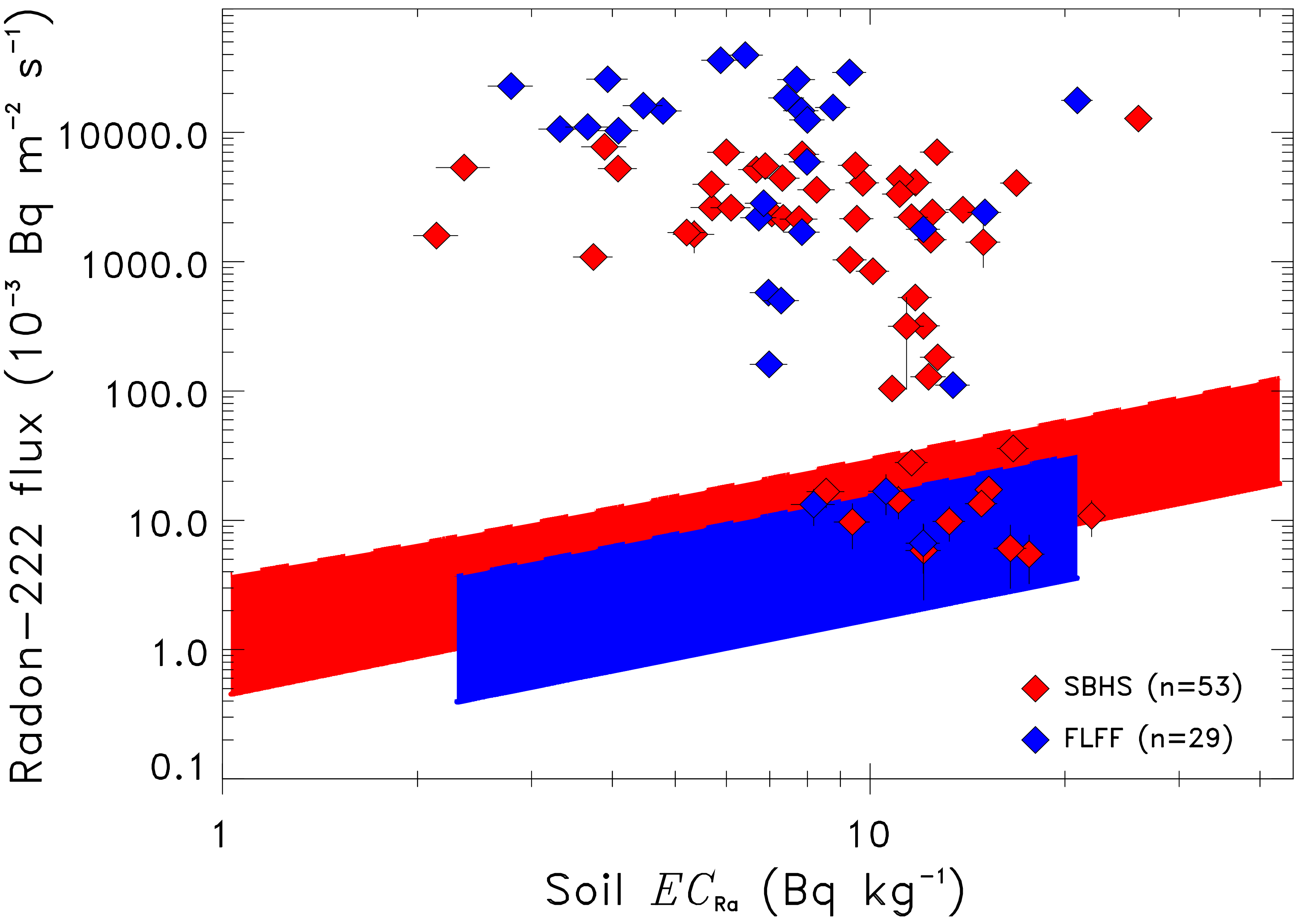

Figure S10. Radon flux as a function of soil EC_{Ra} for both sites from the purely diffusive model of radon transport. Data are shown as diamonds (in blue for FLFF and in red for SBHS). Model calculations are shown as coloured bands accounting for the range of values of data and model parameters: for FLFF, $2.3 \leq EC_{Ra}$ of the soil zone ≤ 20.9 Bq kg^{-1}; for SBHS, $1.0 \leq EC_{Ra}$ of the soil zone ≤ 43 Bq kg^{-1}.

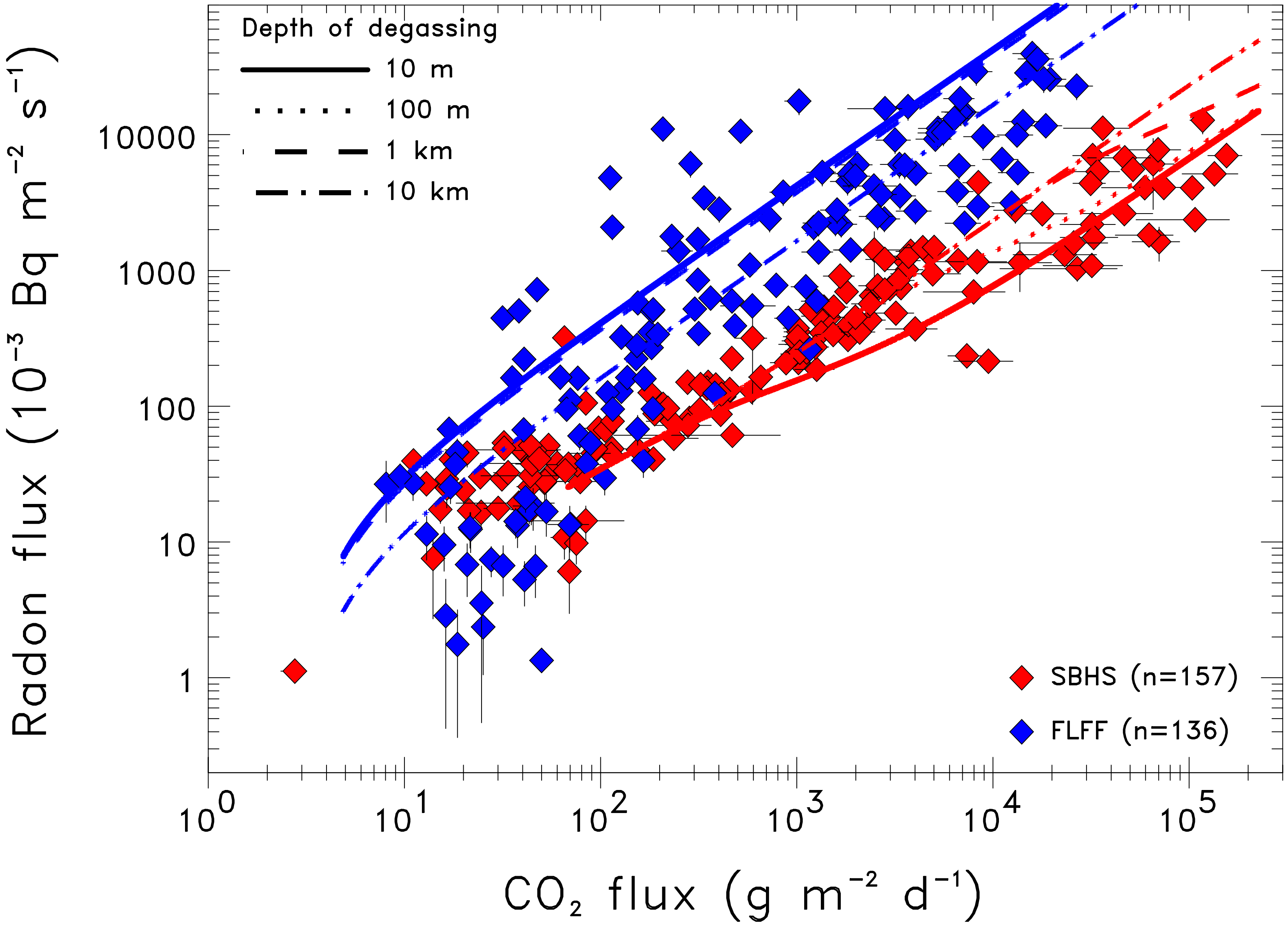

Figure S11. Radon–CO_2 fluxes correlation for both sites from the advective-diffusive model of radon transport. Data are shown as diamonds (in blue for FLFF and in red for SBHS). Model calculations are shown as solid, dotted, dashed, and dash-dot curves depending on the depth of the radon source, *i.e.*, 10 m, 100 m, 1 km, and 10 km, respectively.

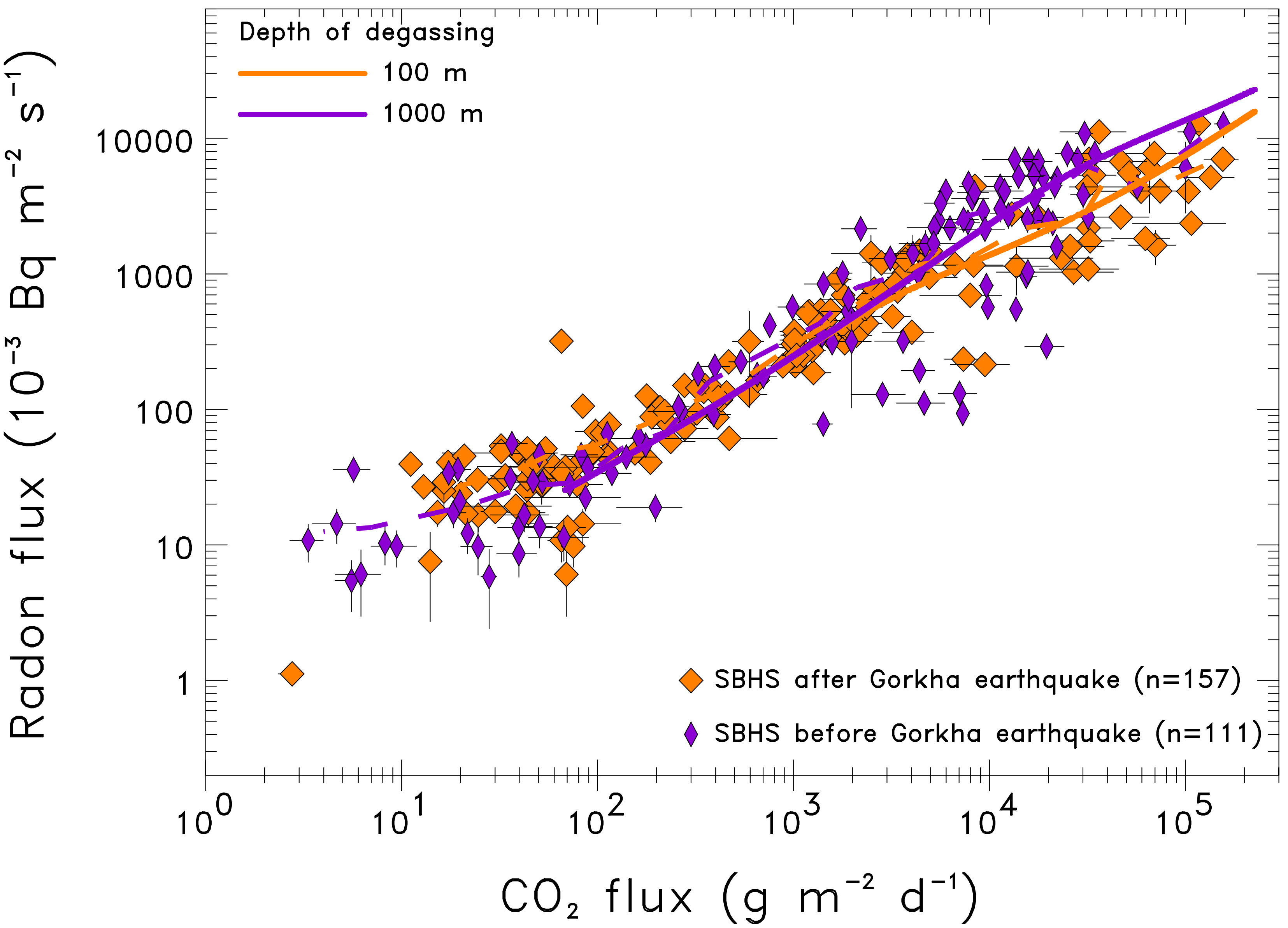

Figure S12. Radon–CO_2 fluxes correlation for SBHS from the advective-diffusive model of radon transport. Data are shown as diamonds, whose colour depends on the measurement period: in purple before the 2015 Gorkha earthquake, and in orange after. Averages of the data are plotted as dashed curves. Model calculations are shown as solid curves, for a radon source depth of 1,000 m (purple) and for a radon source depth of 100 m (orange).
